# Oligodendroglial excitability mediated by glutamatergic inputs and Nav1.2 activation

**DOI:** 10.1038/s41467-017-00688-0

**Published:** 2017-09-15

**Authors:** Emmanuelle Berret, Tara Barron, Jie Xu, Emily Debner, Eun Jung Kim, Jun Hee Kim

**Affiliations:** Department of Cellular and Integrative Physiology, University of Texas Health Science Center, San Antonio, Texas 78229 USA

## Abstract

Oligodendrocyte (OL) maturation and axon-glial communication are required for proper myelination in the developing brain. However, physiological properties of OLs remain largely uncharacterized in different brain regions. The roles of oligodendroglial voltage-activated Na^+^ channels (Na_v_) and electrical excitability in relation to maturation to the myelinating stage are controversial, although oligodendroglial excitability is potentially important for promoting axon myelination. Here we show spiking properties of OLs and their role in axon-glial communication in the auditory brainstem. A subpopulation of pre-myelinating OLs (pre-OLs) can generate Na_v_1.2-driven action potentials throughout postnatal development to early adulthood. In addition, excitable pre-OLs receive glutamatergic inputs from neighboring neurons that trigger pre-OL spikes. Knockdown of Na_v_1.2 channels in pre-OLs alters their morphology, reduces axon-OL interactions and impairs myelination. Our results suggest that Na_v_1.2-driven spiking of pre-OLs is an integral component of axon-glial communication and is required for the function and maturation of OLs to promote myelination.

## Abstract (web summary)

Axon-glial communication is important for myelination. Here the authors show that during postnatal development in rats, a subpopulation of pre-myelinating oligodendrocytes in the auditory brainstem receive excitatory inputs and can generate Na_v_ 1.2-driven action potentials, and that such process promotes myelination.

## Introduction

Oligodendrocytes (OLs) produce the layered myelin sheath surrounding axons, which is essential for fast propagation of saltatory nerve impulses and maintenance of axon integrity in the central nervous system (CNS). OL lineage cells mature to the myelinating stage by proliferating and differentiating from the precursor stage. It has been proposed that the excitability of oligodendroglia, once considered non-excitable cells, is a potentially important mechanism for promoting axon myelination. However, the extent of OL excitability remains controversial. OL lineage cells share some characteristics with neurons, including the expression of functional voltage-activated Na^+^ channels (Na_v_), the ability to generate a spike and the presence of synaptic inputs^1–5^. The synaptic input from neurons can be glutamatergic or GABAergic, but either can induce depolarization^1–7^. However, the ability of oligodendroglia to fire action potentials (APs) exhibiting a distinct spike threshold and repetitive firing is controversial. One recent study demonstrated that OL precursor cells (OPCs) expressing neural/glial antigen 2 (NG2) exhibit a form of repetitive AP firing^2^; however, others found that OPCs display single spike-like events upon depolarizing current injection, or they do not generate any spikes^4, 8^. Furthermore, it has been thought that glial excitability is restricted to OPCs and is rapidly downregulated during the transition from OPCs to immature pre-myelinating OLs (pre-OLs^4, 8^).

Pre-OLs express 2′,3′-cyclic nucleotide phosphohydrolase (CNPase), myelin proteolipid protein and its alternatively spliced isoform DM-20 (DM20-PLP) and OL marker O1, but not NG2^9^. Morphologically, pre-OLs exhibit a number of processes attached to axons and form a few thin sheaths similar to the T-shape morphology described by Bakiri et al. (2011) and Kukley et al. (2010)^8, 10^. Interestingly, pre-OLs are rarely observed in the hippocampus, where OPCs and myelinating OLs are frequently observed^8^, raising the possibility that pre-OLs temporally appear and rapidly mature to myelinating OLs in gray matter areas of the CNS. It remains unknown whether oligodendroglial excitability in the precursor stage is completely lost or can be transferred to pre-OLs in highly myelinated areas of the brain.

In this study, we investigate OL excitability and the constitutive roles of OL beyond the precursor stage through postnatal development in the rat brainstem, where compact myelination is critical for ensuring the fidelity and reliability of neurotransmission^11, 12^. We chose the medial nucleus of the trapezoid body (MNTB) in the auditory brainstem as a highly myelinated and synapse-rich area, where different types of cells (neurons, astrocytes and OLs) are clearly detectable by their shape, size and intrinsic properties: calyx of Held terminals (cup-shaped structures enveloping the postsynaptic cell body^13, 14^), MNTB principal neurons (large and globular, diameter >20 µm, capacitance >30 pF^15^), and OLs (small and round cell body, diameter <10 µm, capacitance <20 pF).

We describe a subpopulation of pre-OLs exhibiting glutamatergic inputs, Na_v_ currents and APs. We further demonstrate that downregulation of Na_v_1.2-driven excitability in these excitable pre-OLs alters the morphological maturation of OL lineage cells, the formation of axon-oligodendroglia interactions and myelination in the auditory brainstem. Our results suggest that oligodendroglial excitability driven by Na_v_1.2 currents is a conserved property during postnatal development and plays an important role in the interactions between oligodendroglial cells and neighboring axons, as well as in myelination.

## Results

### A subpopulation of immature OLs can generate Aps

**Fig. 1:**
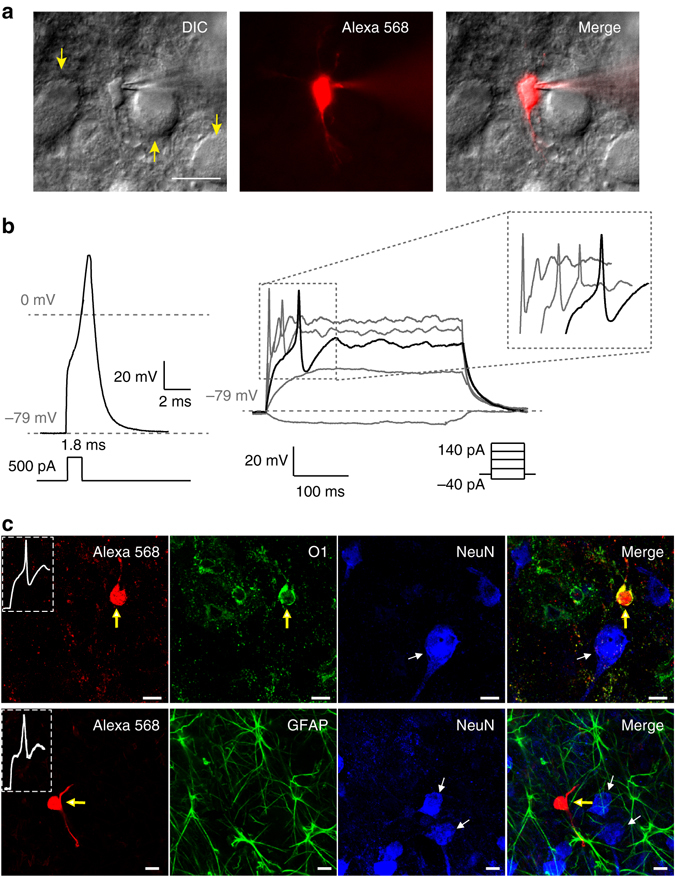
A subpopulation of MNTB OLs can fire APs. **a** Excitable OLs in the MNTB were subjected to whole-cell patch-clamp recording and were filled with Alexa 568 (*red*) during recording for post-recording immunostaining. Differential interference contrast (DIC) image of an excitable OL, which was loaded with Alexa 568 (*red*). *Yellow arrows* indicate MNTB principal neurons. *Scale bar*, 20 μm **b** A single AP was generated by a brief current injection (500 pA for 1.8 ms, *left*) in an excitable OL at P11. APs were evoked by step-current injections from −40 to 140 pA (300 ms, *right*) in the same cells. *Inset*, expanded time scale of area within the dashed box. **c** *Upper* Excitable OLs were immunostained for O1 (OL marker; *green*) and NeuN (neuronal marker; *blue*). A representative excitable OL displayed APs (inset) and expressed O1 (*yellow arrow*) but not NeuN (*white arrow*). *Lower* Excitable OLs were immunostained for GFAP (astrocyte marker; *green*) and NeuN (*blue*). A representative OL expressed neither GFAP nor NeuN (*white arrow*). *Scale bar*, 10 μm

Between postnatal days 7 and 14 (P7−P14), when axon myelination occurs in the auditory brainstem, we identified glial cells that were able to generate APs in response to current injections (Fig. 1a, b). These excitable glial cells were identified as OLs based on post-recording immunostaining, morphology and electrophysiological properties. Cells were filled with Alexa 568 during whole-cell recording and subsequently stained with antibodies against the OL marker O1, the neuronal marker NeuN and the astrocyte marker glial fibrillary acidic protein (GFAP; Fig. 1c). Morphologically, OLs were distinguishable from pre- and postsynaptic neurons and astrocytes based on their shape and size. The O1+ immature OLs had a smaller round cell body of diameter ~10 µm, whereas the presynaptic calyx terminal had a cup-shaped structure enveloping the NeuN+ postsynaptic MNTB principal neuron with a diameter of >20 µm. In addition, the firing properties of excitable OLs were distinct from those of presynaptic terminals and postsynaptic MNTB neurons. Astrocytes with GFAP+ processes did not show spiking properties (Supplementary Fig. 1). The spike-generating excitable glia could thus be identified as OLs, not neurons or astrocytes.

The resting membrane potential of excitable OLs was −73 ± 1.3 mV (*n* = 34). The minimum current required to elicit a single AP was 262 ± 31.9 pA for 1.8 ± 0.2 ms (*n* = 13), and the AP threshold was −32 ± 6.7 mV (*n* = 13), as determined by the membrane potential at the inflection point. A short depolarizing current injection (500 pA, 1.8 ms) evoked a single AP with amplitude 119 ± 22.1 mV and half-width 1.4 ± 0.13 ms in excitable OLs. APs overshot beyond 0 mV, with the peak reaching 29 ± 4.9 mV (*n* = 13, Fig. 1b). Some excitable OLs discharged two to three APs with continued depolarization (300 ms), whereas most showed a tendency for strong adaptation. On the basis of these parameters, the APs of excitable pre-OLs are bona fide APs.

### Excitable OLs are immature pre-OLs beyond the OPC stage

**Fig. 2:**
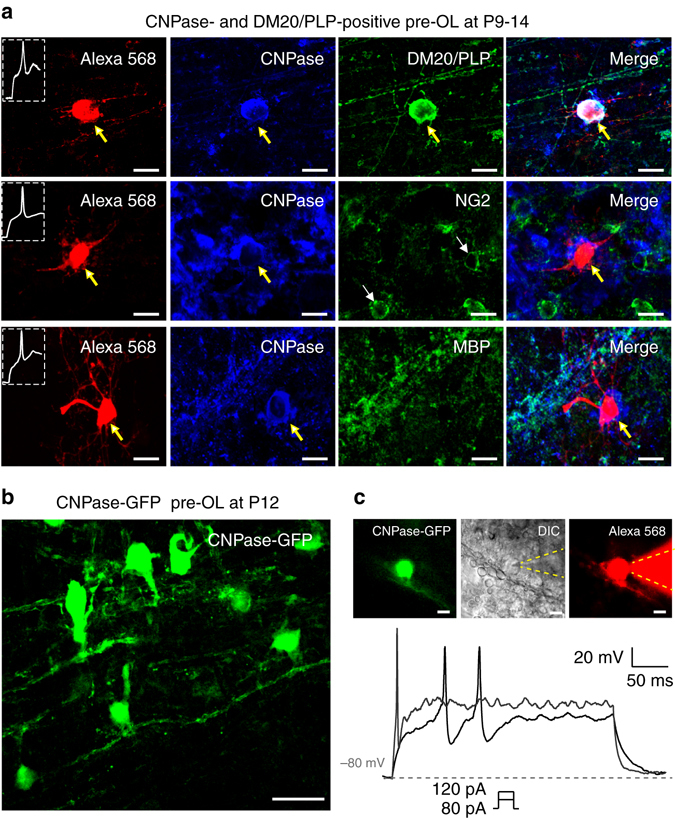
Excitable OLs belong to the pre-myelinating OL stage. **a** Post-recording immunostaining of Alexa 568-filled excitable OLs (*red*) for CNPase (*blue*) and DM20-PLP (*green*, *upper*), NG2 (*green*, *middle*), or MBP (*green*, *lower*) in the MNTB (P9−P14). **b** Confocal image of CNPase-GFP+ cells in the MNTB that were infected by adenovirus encoding pAV.ExSi-CNPase promoter-eGFP. **c** CNPase-GFP+ pre-OLs in the MNTB. A CNPase-GFP+ pre-OL was filled with Alexa 568 during whole-cell recording, as demonstrated in differential interference contrast (DIC) and fluorescence images. Dashed lines indicate the patch pipette. The CNPase-GFP+ pre-OL fired APs in response to step-current injections of 80 and 120 pA (300 ms). *Scale bar*, 10 μm

Next, we determined the developmental stage of the excitable OLs. We performed post-recording immunostaining of excitable OLs using specific markers for each stage: NG2 for OPCs, CNPase and DM20-PLP for pre-OLs and myelin basic protein (MBP) for mature myelinating OLs^8, 9^. Excitable OLs were positive for CNPase and DM20-PLP but not for NG2 or MBP (at P9−P14, *n* = 10, 7, 7 and 5, Fig. 2a). CNPase immunoactivity was detected from processes and somatic membranes, rather than the somatic cytosol of pre-OLs (Supplementary Fig. 2). To confirm the identity and differentiation status of these excitable OLs, we injected an adenovirus encoding CNPase promoter-eGFP into the MNTB using stereotaxic injection (Fig. 2b). A subpopulation of CNPase-eGFP+ cells (8 out of 32 recorded cells) generated APs in response to depolarizing current injections (80 and 120 pA, 300 ms; Fig. 2c). Thus, excitable OLs belong to the immature pre-myelination stage (i.e., pre-OL).

### Morphological and functional characterization of CNPase +OLs

**Fig. 3:**
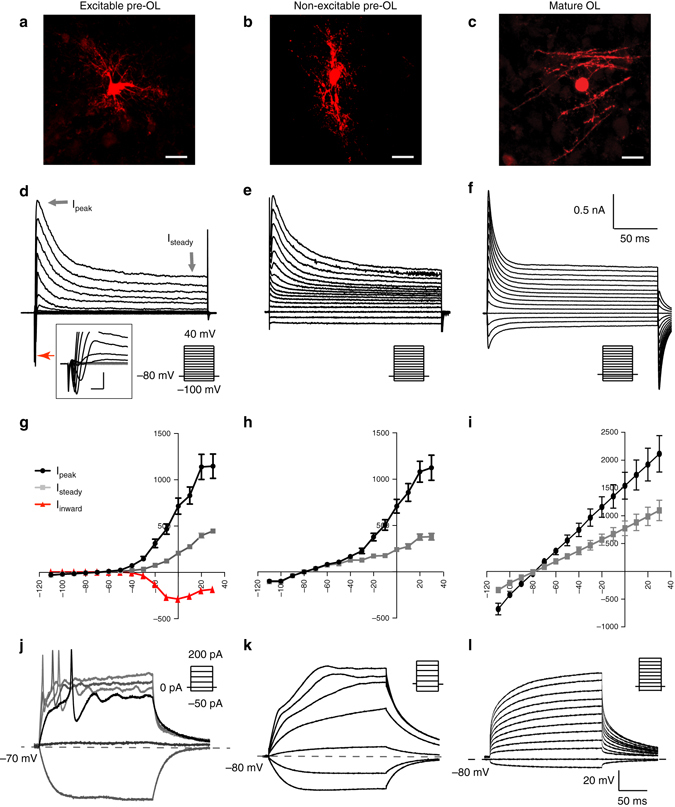
Morphology and physiological properties of excitable vs. non-excitable pre-OLs and mature OLs. **a**–**c** Confocal images of excitable pre-OLs **a**, non-excitable pre-OLs **b**, and mature OLs **c**, which were filled with Alexa 568 during whole-cell recordings. *Scale bar*, 10 μm. **d**–**f** Representative traces of Na_v_-mediated currents generated by voltage steps (from –100 to 40 mV for **d** and **e**, from –110 to 30 mV for **f**) in excitable pre-OLs, non-excitable pre-OLs and mature OLs. *Gray* *arrows* indicate both peak outward currents (I_peak_) and steady-state outward currents (I_peak_), and the *red arrow* indicates inward currents. *Inset*, expanded time scale of inward currents from excitable pre-OLs (scale; 2 ms and 400 pA, **d**). **g**–**i** Current–voltage (*I*–*V*) relationship for I_peak_ (*black*), I_steady_ (*gray*), and I_inward_ (*red*) from excitable pre-OLs **g**. I-V relationship for I_peak_ (*black*) and I_steady_ (*gray*) from non-excitable pre-OLs **h** and mature OLs **i**. Data represent the mean ± s.e.m. **j** Excitable pre-OLs fired APs in response to current injections (>50 pA, 300 ms) in current-clamp mode. **k, l** Recordings of membrane potential from a non-excitable pre-OL **k** and a mature OL **l** in response to current injections (300 ms, from –80 to 200 pA for **k**, and –50 to 400 pA for **l**). *Dashed lines* indicate the resting membrane potential

In the MNTB (P7–14), we classified three different types of CNPase+ OLs based on their structural and functional properties. In the morphological analysis of three-dimensional (3D) reconstructed images, excitable pre-OLs displayed a soma diameter of 9.7 ± 0.67 μm, a cell volume of 1441 ± 441.5 μm^3^, and a total process length of 697 ± 72.6 μm (*n* = 12). Their main processes evidently contacted axons or neurons but did not align with or enwrap axons, consistent with immature OLs (Fig. 3a and Supplementary Fig. 3). Non-excitable pre-OLs had a soma diameter of 12.1 ± 2.4 µm (*n* = 15), and a number of processes had not yet enwrapped axons. The cell volume was 2593 ± 290.5 μm^3^, and the total process length was 784 ± 85.1 μm (*n* = 4, Fig. 3b and Supplementary Fig. 3). Thus, the morphology of non-excitable pre-OLs was consistent with that of immature pre-OLs not yet starting to myelinate, similar to excitable pre-OLs. However, non-excitable pre-OLs exhibited a larger membrane capacitance compared with excitable pre-OLs (40 ± 2.5 pF, *n* = 34, vs. 17 ± 0.9 pF, *n* = 26; *t-*test, *P* < 0.0001, Supplementary Fig. 3), which may be due to a larger cell volume. In addition, we identified CNPase+ mature OLs, which formed thin enwrapping structures around axons and exhibited larger membrane capacitance (>80 pF, Fig. 3c) and lower input resistance compared with excitable and non-excitable OLs (mature OLs: 0.28 ± 0.07 GΩ, *n* = 6, non-excitable: 0.49 ± 0.32 GΩ, *n* = 10 and excitable: 0.9 ± 0.1 GΩ, *n* = 18, analysis of variance (ANOVA), *P* = 0.001, Supplementary Fig. 3).

Excitable pre-OLs exhibited Na_v_-mediated inward currents, which were completely blocked with tetrodotoxin (TTX; 1 μM), indicating the presence of Na_v_ channels. The current–voltage relationship curve (*I*–*V* curve) revealed outwardly rectifying K^+^ currents and voltage-activated inward currents with an inverted bell shape (290 ± 27.2 pA at 0 mV, *n* = 32, Fig. 3d, g). Non-excitable pre-OLs displayed the fast inactivating outward currents, similar to those seen in excitable OLs, but lacked distinctive voltage-activated inward currents (Fig. 3e, h). The *I*–*V* curve, showing outward rectification, was similar to that obtained from excitable cells. Mature OLs had larger outward currents with less rectification (Fig. 3f, i) and also had Ba^2+^-sensitive inwardly rectifying K^+^ currents, the upregulation of which has been reported during OL maturation^16^. In contrast, excitable pre-OLs did not display Ba^2+^-sensitive inwardly rectifying K^+^ currents (Supplementary Fig. 3).

Excitable pre-OLs generated APs in response to depolarizing current injection (Fig. 3j), whereas the membrane potential of non-excitable OLs and mature OLs increased with increasing current injections, not showing APs (Fig. 3k, l). Non-excitable pre-OLs and mature OLs displayed a resting membrane potential of −81 ± 0.6 mV (*n* = 32) and –82 ± 1.8 mV (*n* = 6), respectively, which were more negative than those obtained with the excitable pre-OLs (Supplementary Fig. 3).

### Comparison of excitable pre-OLs and OPCs

**Fig. 4:**
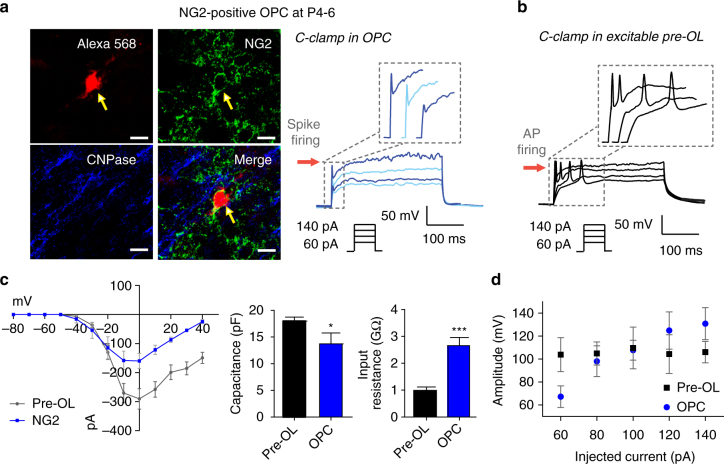
Physiological properties of excitable pre-OLs differ from those of OPCs. **a** Post-recording immunostaining of an Alexa 568-filled OPC (*red*) expressing NG2 (*green*) but not CNPase (*blue*). *Scale bars*, 10 µm. The same OPC displayed depolarizing spikes in response to current injections (60–140 pA, 300 ms) in current-clamp recordings. *Inset*, expanded time scale of the area within the *dashed box*. **b** An excitable pre-OL (P11) exhibiting distinct APs in response to current injections (60–140 pA, 300 ms). *Inset*, expanded time scale of the area within the dashed box. **c** *I*–*V* relationship of I_inward_ in an OPC (*blue*) and a pre-OL (*black*) and comparison of passive properties (capacitance and input resistance). **d** Relationship between spike amplitude and amount of current injected in OPCs and pre-OLs. Data represent the mean±s.e.m. *P < 0.05, ***P < 0.0001

OPCs (NG2+ cells) have voltage-activated Na^+^ currents^1–3^, but it is unclear whether these currents can generate spikes. We thus compared the active and passive properties of pre-OLs (CNPase+/DM20-PLP+) with those of OPCs (NG2+) in the MNTB. OPCs in the MNTB at P4−P6 could be classified into two populations based on the presence or absence of Na_v_-mediated currents. In response to current step injections (60–140 pA/300 ms), OPCs with Na^+^ currents displayed graded depolarization peaks without a distinct threshold. A sharp depolarization was followed by a small and incomplete repolarization, and then a large graded depolarization. OPCs did not show a substantial overshot beyond 0 mV or repetitive firing (Fig. 4a). These properties differed significantly from pre-OL spikes, which displayed a distinct threshold and a depolarization peak reaching ~30 mV (an all-or-none spike), and often exhibited repetitive firing (Fig. 4b). OPCs had a smaller Na^+^ current (159 ± 26 vs. 290 ± 27.2 pA at 0 mV, *n* = 6 and 32, respectively, at 0 mV), smaller capacitance (14 ± 2.0 vs. 17 ± 0.7 pF, *n* = 6 and 26, respectively; *t*-test, *P* *=* 0.019), and larger input resistance (2.6 ± 0.3 vs. 0.9 ± 0.1 GΩ, *n* = 6 and 18, respectively; *t*-test, *P* *<* 0.0001; Fig. 4c). In addition, the spike amplitude in OPCs gradually increased in response to larger current injections, whereas the spike amplitude in pre-OLs remained constant (Fig. 4a, b, d). Both pre-OLs and OPCs exhibited Na_v_-mediated currents, but their passive and active properties differed significantly.

### Na_v_1.2-mediated Na^+^ currents drive APs in excitable pre-OLs

**Fig. 5:**
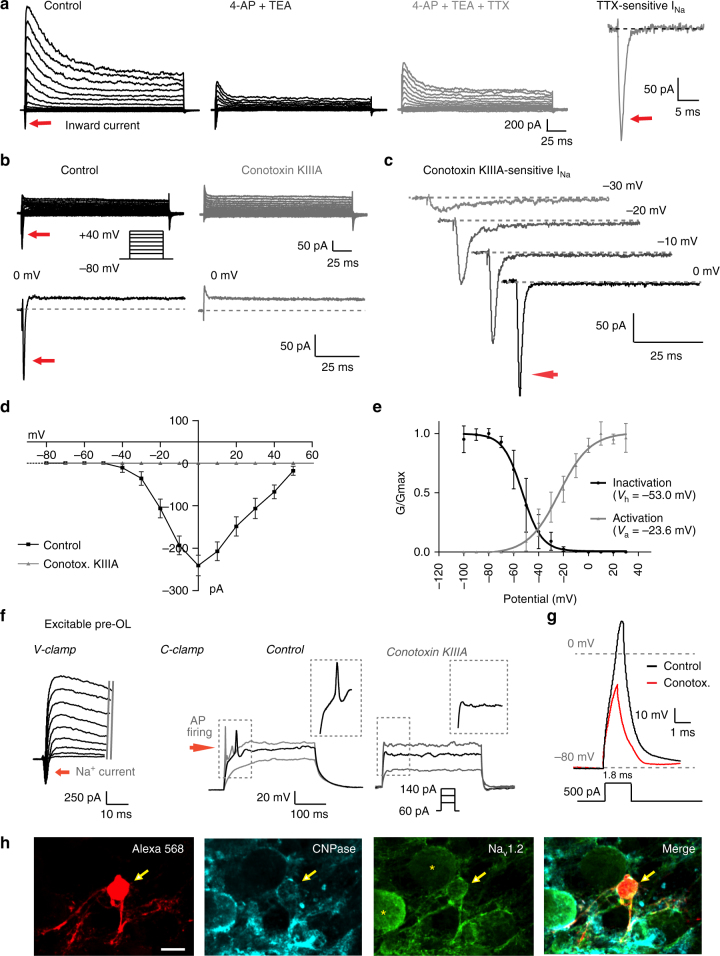
Na_v_1.2-mediated Na^+^ currents underlie the excitability of pre-OLs and allow generation of APs. **a** Representative traces of voltage-activated inward and outward Na_v_-mediated currents in response to step-voltage commands (−80 to 40 mV, holding at −80 mV) in excitable pre-OLs (P9) in control conditions (*left*), in the presence of 2 mM 4-AP and 10 mM TEA (*middle*), and with addition of 1 µM TTX (*right*). *Arrows* indicate peak outward currents (I_peak_), steady-state outward currents (I_steady_), and inward currents. The TTX-sensitive current was obtained by subtracting the inward current in the 4-AP+TEA condition from that in the TTX condition. **b** I_Na_ in excitable pre-OLs in response to step-voltage commands (as in **a**) in the presence of 2 mM 4-AP, 10 mM TEA, and 0.2 mM CdCl_2_ and with a Cs-based internal solution. µ-Conotoxin KIIIA (1 µM; Na_v_1.2 channel inhibitor) completely inhibited I_Na_. *bottom* Expanded time scale for the inward currents at 0 mV. **c** Representative traces of µ-conotoxin KIIIA–sensitive I_Na_ at −30, −20, −10 and 0 mV *top* to *bottom*. **d** Current–voltage relationship of I_Na_ in control conditions (*black*) and in the presence of µ-conotoxin KIIIA (*gray*). **e** Mean voltage dependence of activation and inactivation for I_Na_. Inactivation and activation kinetics of Na_v_ in pre-OLs were examined by a series of 200-ms prepulses (−100 to −10 mV) followed by a 10-ms test pulse at 0 mV. *V*_a_ and *V*_h_ indicate the half-activation and half-inactivation potentials, respectively, of Na_v_ in pre-OLs. Data represent the mean ± s.e.m. **f** Voltage-activated K^+^ and Na^+^ currents in response to step depolarization (−60 mV to 30 mV) in excitable pre-OLs in voltage-clamp recordings (P12). Pre-OLs displayed APs in response to current injections (60–140 pA). In the same cells, µ-conotoxin KIIIA (1 µM) completely inhibited AP firing. Inset, magnified traces of APs. **g** A single AP evoked by a brief current injection (500 pA, 1.8 ms) in control conditions (*black*) and in the presence of µ-conotoxin KIIIA (1 µM, *red*). **h** Post-recording immunostaining for CNPase (*cyan*) and Na_v_1.2 (*green*) in the same cell as **f** loaded with Alexa 568 (*red*). Arrows indicate a pre-OL that displayed I_Na_ and APs during whole-cell recording. *Asterisks* indicate MNTB principal neurons (diameter >20 µm). *Scale bar*, 10 μm

The transient peak and sustained K^+^ currents in excitable pre-OLs were 2.2 ± 0.42 and 0.6 ± 0.03 nA, respectively, in control conditions (*n* = 9). Concomitant application of 2 mM 4-aminopyridine (4-AP) and 10 mM tetraethylammonium chloride (TEA-Cl) decreased the transient peak and sustained components to 0.6 ± 0.08 and 0.4 ± 0.09 nA, respectively (*n* = 6; *P* *<* 0.001, Fig. 5a). In addition, excitable pre-OLs expressed a fast-activating and inactivating TTX-sensitive Na^+^ current but not a non-inactivating persistent Na^+^ current (Fig. 5a). Thus, excitable pre-OLs displayed 4-AP-sensitive K^+^ currents, sustained TEA-sensitive K^+^ currents, and TTX-sensitive Na^+^ currents underlying their AP firing.

To identify the Na_v_ channel subtypes expressed in excitable pre-OLs, we examined their pharmacological and biophysical properties. We recorded voltage-activated Na^+^ currents (I_Na_) using Cs-methanesulphonate internal solution in the presence of 4-AP (2 mM), TEA (10 mM) and cadmium (Cd, 200 µM, a voltage-gated Ca^2+^ channel inhibitor). I_Na_ recorded in excitable pre-OLs were sensitive to µ-conotoxin KIIIA, an inhibitor of Na_v_1.2 channels in the CNS^17, 18^ (Fig. 5b). We next assessed the inactivation and activation kinetics of these currents, as this is one criteria used to classify Na_v_ subtypes^19, 20^. The µ-conotoxin KIIIA–sensitive I_Na_ were activated and inactivated in a voltage-dependent manner from −40 to +40 mV, with a peak amplitude of 241 ± 24.1 pA at 0 mV (*n* = 11, Fig. 5c, d). In an inactivation curve fit by the Boltzmann function, the half-values of the voltage dependence of activation and inactivation were *V*_a_ = −23.6 ± 3.2 mV (*n* = 7) and *V*_h_ = −53.0 ± 4.2 mV (*n* = 10, Fig. 5e), respectively. These results were similar to values previously reported for the Na_v_1.2 channel subtype in excitable cells (*V*_a_ = −24 mV, *V*_h_ = −53 mV^20^).

In excitable pre-OLs, µ-conotoxin KIIIA completely inhibited Na^+^ currents and AP firing (Fig. 5f, g), suggesting that the I_Na_ mediated by Na_v_1.2 channels underlies pre-OL excitability. Post-recording immunostaining revealed that these excitable pre-OLs expressed Na_v_1.2 and CNPase (Fig. 5h). CNPase+ pre-OLs predominantly expressed Na_v_1.2 in their somatic membrane and processes, which are often apposed to neuronal soma, but did not express other subtypes of Na_v_ such as Na_v_1.6, expressed exclusively in the axons of MNTB neurons (Supplementary Fig. 4). Taken together, these results indicated that Na_v_1.2 is the major Na_v_ subtype that is functionally expressed and required for firing APs in excitable pre-OLs.

### Axonal glutamate triggers spikes in excitable pre-OLs

**Fig. 6:**
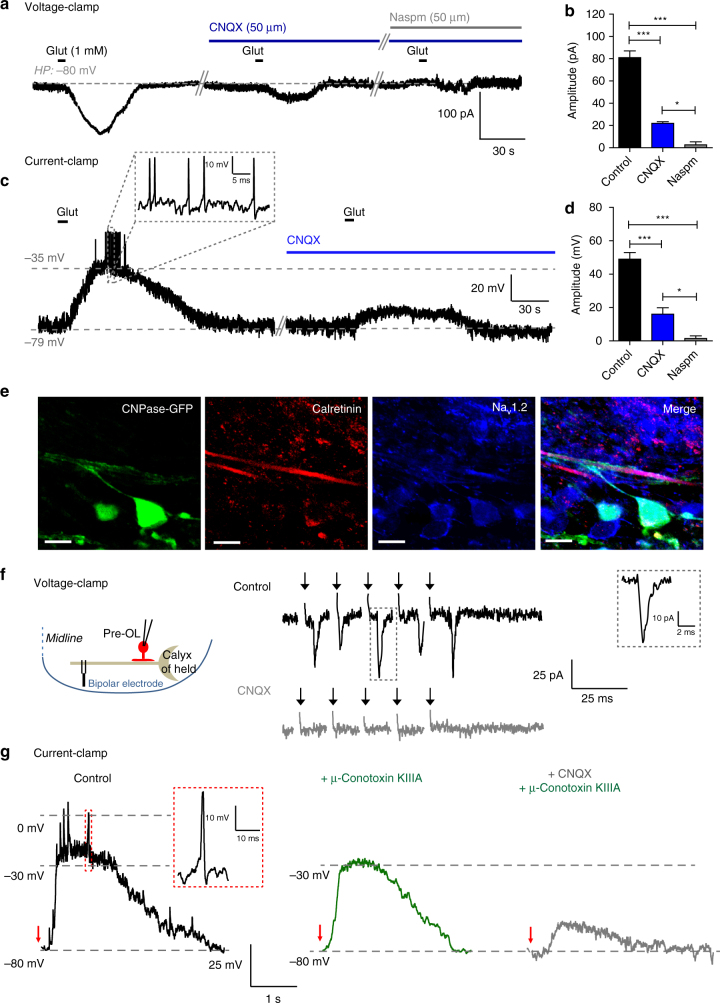
AMPA-mediated currents evoked by neuron activity drive AP firing in pre-OLs. **a** Representative trace of synaptic currents evoked by glutamate application (1 mM) in an excitable pre-OL during voltage-clamp recording (P10). CNQX (50 µM; AMPAR blocker) largely reduced the synaptic current; additional application of Naspm (50 µm; Ca^2+^-permeable AMPAR blocker) abolished most of the remaining current. **b** Summary of glutamate receptor-mediated synaptic currents in the presence of CNQX and Naspm. **c** Representative trace of glutamate-evoked depolarization and firing in an excitable pre-OL in a current-clamp recording. Application of CNQX blocked nearly all depolarization and subsequent spikes. *Inset*, expanded time scales of APs. **d** Summary of membrane potential changes upon glutamate application in the presence of CNQX and Naspm. Data represent the mean ± s.e.m.; *P < 0.05, ***P < 0.0001. **e** Immunostaining of CNPase-eGFP+ cells with antibodies against calretinin (Ca^2+^-bind protein as a marker for axon fibers, *red*) and Na_v_1.2 (*blue*) in the MNTB (P14). Note that processes of CNPase-eGFP+ cells make contact with axon fibers. *Scale bar*, 10 μm. **f** Glutamate-mediated synaptic currents evoked by axon fiber stimulation (100 Hz, 1 s; see *diagram*) in an excitable pre-OL (P11). CNQX blocked the synaptic current. **g** Depolarization and firing activity evoked by electrical stimulation (100 pulses at 100 Hz) in a current-clamp recording of an excitable pre-OL. Inset, expanded time scales of an AP indicated in the box. Application of μ-conotoxin KIIIA (1 μM) completely blocked firing activity, and additional application of CNQX (50 μM) significantly reduced depolarization. *Dashed lines* indicate membrane potential. *Red arrows* indicate the end time point of the afferent fiber simulation

We next sought to understand how Na_v_1.2 channels generate spikes in excitable pre-OLs under physiological conditions. We found that excitable pre-OLs have functional AMPA receptors (AMPARs) and glutamate-mediated currents. In voltage-clamp recordings of excitable pre-OLs, local glutamate application (1 mM) triggered an inward current (80.9 ± 10.6 pA; *n* = 5) that was significantly reduced in the presence of CNQX (AMPAR blocker, 100 μM; 21.9 ± 2.4 pA; *n* = 5; ANOVA, *P* < 0.0001). The remaining inward current was completely abolished by additional application of the Ca^2+^-permeable AMPAR antagonist 1-Naphthyl acetyl spermine trihydrochloride (Naspm, 2.6 ± 4.5 pA; *n* = 5; ANOVA, *P* < 0.0001, Fig. 6a, b), indicating that excitable pre-OLs have both Ca^2+^-permeable and Ca^2+^-impermeable AMPAR. Next, we tested whether these glutamate-mediated currents could depolarize pre-OLs to reach spike threshold. In current-clamp recordings at approximately −80 mV, local glutamate application depolarized pre-OLs by 49.0 ± 7.8 mV (from −80 mV to approximately –35 mV, *n* = 5) and initiated a spike burst (Fig. 6c). Both the depolarization and the spike bursts were inhibited by CNQX (depolarization by 16.0 ± 7.8 mV; *n* = 4; ANOVA, *P* < 0.0001) and additional application of Naspm (1.5 ± 3.0 mV; *n* = 4; ANOVA, *P* < 0.0001, Fig. 6c, d). These results demonstrated that AMPAR-dependent glutamate-mediated currents could depolarize excitable pre-OLs enough to fire spikes.

Based on this observation, we next asked where glutamate originates to stimulate pre-OLs under physiological conditions. We found that pre-OLs in the MNTB formed a synaptic interaction with surrounding axons and received glutamatergic inputs from axons. Excitable pre-OLs (CNPase-eGFP+/Na_v_1.2+ cells) had thin processes that attached to and aligned with nearby axon fibers (Fig. 6e). Axonal stimulation evoked AMPAR-mediated excitatory postsynaptic currents (32.3 ± 11.4 pA; *n* = 8 cells). These synaptic currents were completely blocked by CNQX (Fig. 6f), indicating that excitable pre-OLs receive glutamatergic synaptic inputs from surrounding axons during neuronal activity. In current-clamp recordings, axon fiber stimulation (100 Hz, 1 s) resulted in membrane depolarization of pre-OLs from −80 to −30 mV and induced spike firing, followed by a slow return to the resting potential (Fig. 6g). The application of µ-conotoxin KIIIA completely blocked these pre-OL spikes, indicating that the spikes were driven by Na_v_1.2 currents. Slow depolarization induced by axonal simulation was mostly inhibited by CNQX (Fig. 6g), suggesting that neuronal activity-dependent glutamatergic inputs on excitable pre-OLs could induce pre-OL spikes.

### Developmental changes in the excitable pre-OL population

**Fig. 7:**
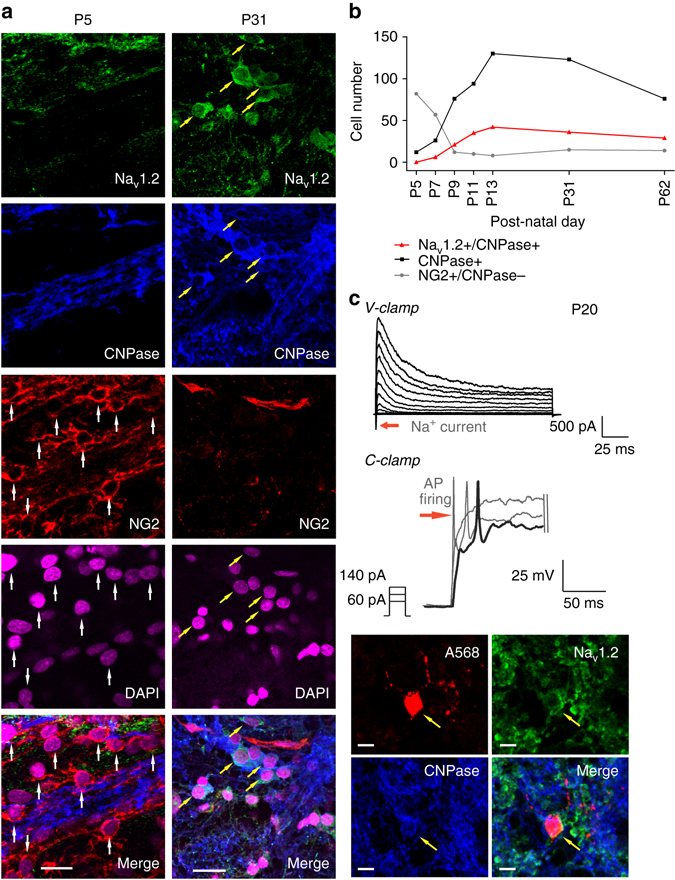
The population of excitable pre-OLs increases during early postnatal development in the MNTB. **a** Immunostaining for Na_v_1.2 channels (*green*), CNPase (*blue*) and NG2 (*red*) as well as DAPI staining (*magenta*) in the MNTB at P5 and P31. *Yellow arrows* indicate CNPase+/Na_v_1.2+ pre-OLs, and *white arrows* indicate NG2+ cells. *Scale bar*, 20 μm. **b** Total number of NG2+/CNPase– cells (*gray*), CNPase+ cells (*black*), and Na_v_1.2+/CNPase+ cells (excitable pre-OLs, *red*) from three slices (from four different brains) each at P5, P7, P9, P11, P13, P31, and P62). **c** Voltage-activated K^+^ and Na^+^ currents in response to step depolarization (−80 to 40 mV) in an excitable pre-OL (*left*) at P20. The same pre-OL exhibited APs in response to current injections (60–140 pA). Post-recording immunostaining revealed that the same cell (loaded with Alexa 568, *red*, *arrow*) expressed CNPase (*blue*) and Na_v_1.2 (*green*). *Scale bar*, 10 μm

To test whether pre-OL excitability is temporally required for initial myelination during postnatal development, we examined changes in excitable pre-OL populations in the MNTB from P5 to P62 using specific markers (*n* = 3 slices in each of five animals per age group). Excitable pre-OLs expressed CNPase and Na_v_1.2 channels (Na_v_1.2+/CNPase+; Fig. 7a). At P5, a number of NG2+ cells (OPCs), but few CNPase+ cells (pre-OLs), were observed in the MNTB. CNPase+/Na_v_1.2+ cells (excitable pre-OLs) started to appear at P7 and constituted ~20–50% of total CNPase+ cells (total pre-OLs) at P9 and P13. As the number of pre-OLs increased from P9 to P13, NG2+ cells (OPCs) were conversely reduced in number. Interestingly, the population of CNPase+/Na_v_1.2+ cells was sustained later in development (P31) and into adulthood (P62; Fig. 7b). Moreover, at P19−P22, CNPase+/Na_v_1.2+cells exhibited a substantial I_Na_ (peak 199 ± 15.7 pA, *n* = 6) as well as AP firing (Fig. 7c). This result indicated that a subpopulation of CNPase+ pre-OLs, displaying functional Na_v_1.2 currents and maintaining their ability to fire APs, is present in the MNTB throughout development and into adulthood.

### Roles of excitable pre-OLs in OL maturation and myelination

**Fig. 8:**
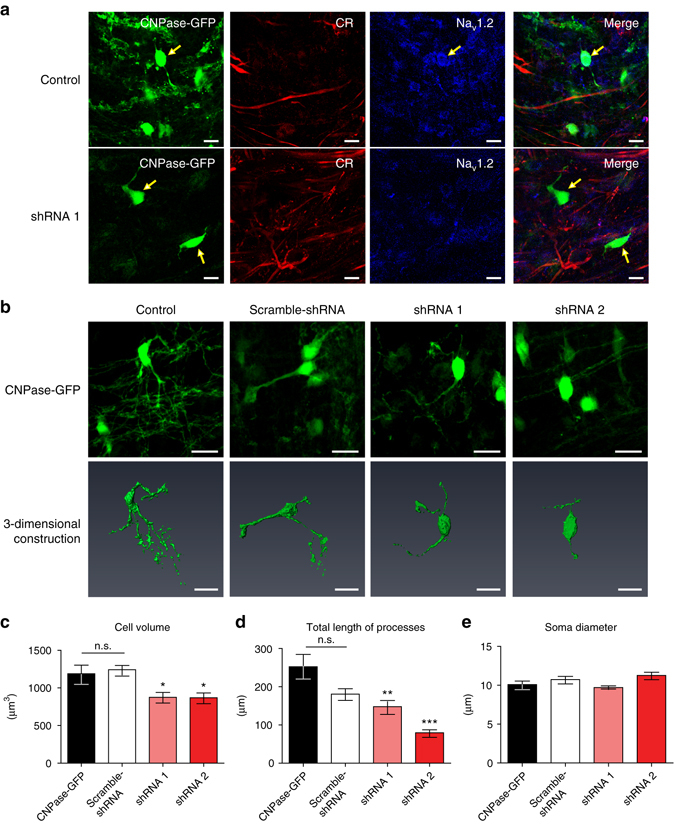
Na_v_1.2 channels are necessary for proper elaboration of pre-OL structures and interaction with axons. **a** Immunostaining of CNPase-GFP+ cells for calretinin (*red*) and Na_v_1.2 (*blue*) in the MNTB (P21) following injection of empty vector (control, *upper*) and vector encoding shRNA**1** for Na_v_1.2 (*lower*). *Yellow arrows* indicate shRNA virus-infected cells. *Scale bar*, 10 μm **b** Representative confocal images (*upper*) and 3D reconstruction (*lower*) of CNPase-GFP+ pre-OLs in control cells and in cells infected with scrambled shRNA, shRNA1, or shRNA2. *Scale bar*, 20 μm. **c**–**e** Summary of the data for cell volume **c**, total length of processes **d**, and soma diameter **e** under all conditions. All data represent the mean ± s.e.m.; *P < 0.05, **P < 0.01, ***P < 0.0001. n.s., nonsignificant difference

To evaluate the function of Na_v_1.2-driven spikes in excitable pre-OLs in the MNTB, we induced a knockdown of the Na_v_1.2 channel specifically in CNPase+ pre-OLs. We used an adenovirus expressing a small hairpin RNA (shRNA) against rat Na_v_1.2 in the CNPase-eGFP vector and injected it into the MNTB. We tested two shRNAs (shRNA1 and 2) against rat Na_v_1.2 with different binding sites to rule out any nonspecific effect of the virus carrying the shRNA. In vector-only controls, 38.8 ± 4.6% of cells were positive for both CNPase-eGFP and Na_v_1.2. In the group injected with shRNA1, the population of double-positive cells was reduced to 9.9% ± 1.2%, indicating an shRNA effectiveness of ~74.3% (Fig. 8a and Supplementary Fig. 5). In addition, we confirmed that the CNPase-eGFP virus restricted GFP expression specifically to OLs, and that the shRNA was under control of the CNPase promoter (Supplementary Fig. 6). The knockdown of Na_v_1.2 did not alter the number of CNPase+cells compared with control, although the knockdown induced significant morphological and structural changes in the pre-OLs. The alignment of pre-OLs surrounding the axons was altered, and the number of processes was reduced (Fig. 8a). The shRNA-infected pre-OLs did not display Na_v_1.2-mediated Na^+^ currents or APs (*n* = 25 cells from shRNA1-infected cells, and *n* = 10 cells from five shRNA2-infected cells, Supplementary Fig. 5). To rule out any shRNA toxicity effect, we used a scrambled shRNA. After 3D reconstruction of CNPase-eGFP+ cells in control, scrambled shRNA, and the two shRNA knockdown models (shRNA1 and 2), we quantified cell volume, soma size and total length of processes. There was no significant difference in these three aspects between control and scrambled shRNA-infected cells, and thus the scrambled shRNA-infected cells had morphological characteristics similar to those of the control. Pre-OLs in which Na_v_1.2 was knocked down (shRNA1 and shRNA2-infected cells) had a smaller cell volume (1188 ± 115.9 µm^3^ in control and 1242 ± 56.9 µm^3^ in scrambled vs. 875.9 ± 65.36 µm^3^ in shRNA1 and 869.6 ± 63.12 µm^3^ in shRNA2; ANOVA, *P* *=* 0.0001, *n* = 24, 27, 25 and 27, respectively, Fig. 8b, c). The lengths of processes were also significantly reduced upon Na_v_1.2 knockdown in both shRNA1 and shRNA2-infected cells (252 ± 31.9 µm and 181 ± 14.2 µm in control and scrambled vs. 147 ± 16.1 µm and 79 ± 7.8 µm in shRNA1 and shRNA2; ANOVA, *P* *=* 0.0001, *n* = 24, 27, 25, and 27, Fig. 8b, d). There was no significant difference in the soma diameter (10 ± 0.4 µm and 11 ± 0.4 µm in control and scrambled vs. 10 ± 0.2 µm and 11 ± 0.4 µm in shRNA1 and shRNA2; ANOVA, *n* = 24, 27, 25 and 27, Fig. 8b, e). Taken together, these results demonstrated that the knockdown of Na_v_1.2 altered the morphological development of pre-OLs, which are in the critical stage preceding myelination.

**Fig. 9:**
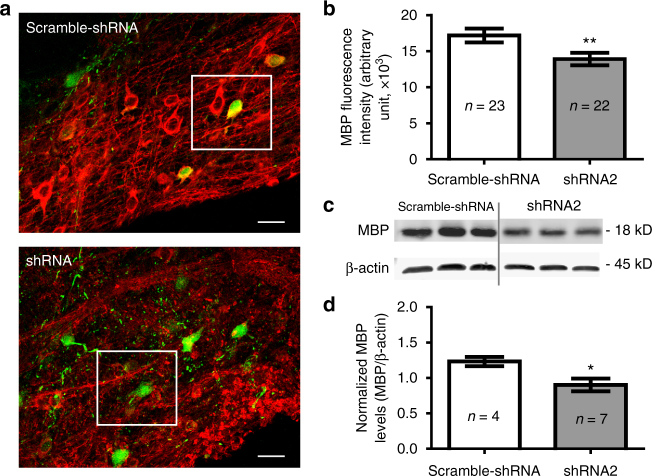
Knockdown of Na_v_1.2 channels in pre-OLs impacts the myelination. **a** Confocal images of immunostaining for MBP in the auditory brainstem following the viral injection of vectors encoding scrambled shRNA (*upper*) and shRNA2 for Na_v_1.2 (*lower*). *Scale bar*, 20 μm. **b** Summary of the fluorescence intensity of MBP (*red*), which was measured in a 50 μm × 50 μm area surrounding CNPase-GFP+ cells within (*white box*). **c** Representative western blots probed for MBP and β-actin levels from three individual brainstem samples in each groups injected with scrambled shRNA (*upper*) and shRNA2. **d** Summary of normalized MBP levels. Detected western blot intensities for MBP were normalized to corresponding β-actin band intensities. All data represent the mean ± s.e.m.; *P < 0.05, **P < 0.01

We thus examined the Na_v_1.2 knockdown effect on myelin production in the brainstem. To quantify the expression level of MBP, we performed immunostaining and western blot in the scrambled shRNA- and shRNA2-infected brainstems. After confocal imaging, the fluorescence intensity of MBP in the local area surrounding of CNPase-GFP+ cells was quantified. Although MBP expression was seen in CNPase-GFP+ cells in both scrambled shRNA and shRNA2-infected brainstems, the intensity of MBP was significantly reduced in the shRNA2-infected brainstem (*n* = 23 individual areas in scrambled vs *n* = 22 in shRNA2, Fig. 9a, b). In addition, western blot analysis demonstrated that MBP level was considerably decreased in shRNA2-infected brainstems compared to scrambled shRNA-infected groups (*n* = 4 in scrambled vs *n* = 7 in shRNA2, Fig. 9c, d). Thus, Na_v_1.2 channels in pre-OLs are potentially involved in the formation of myelination. Overall, our results suggested that functional Na_v_1.2 channels are necessary for proper development of pre-OL processes and elaboration of the connecting structures between pre-OLs and axons, ultimately impacting compact myelination.

## Discussion

Our study demonstrates that a subpopulation of pre-OLs, beyond the OPC stage, display functional Na^+^ currents sufficient to generate APs under physiological conditions in the MNTB of the rat auditory brainstem. Na_v_1.2-mediated excitability is required for pre-OLs to form and extend their processes, which facilitate proper contacts with axons for myelination. These findings indicate that the excitability of pre-OLs is important during OL maturation and axon myelination in the brainstem.

In the rat auditory brainstem, excitable pre-OLs exhibit APs with a discrete threshold of approximately −37 mV, an amplitude of ~110 mV, and an AP peak reaching ~30 mV. All-or-none APs in excitable cells are well characterized, having a threshold near −40 mV, a depolarizing AP peak that approaches the equilibrium potential for Na^+^ (E_Na_; ~50 mV), and a pronounced repolarization^21–24^. On the basis of these criteria, the APs of excitable pre-OLs are bona fide APs, suggesting that a subpopulation of OLs beyond the precursor stage can generate Na^+^ current-mediated APs in the CNS.

NG2+ OPCs have been described as unique in glial development because of their excitability in the white matter and cortex; however, there is little agreement on their ability to fire APs^1–3^. From previous studies, there is a high degree of heterogeneity among OPCs with respect to passive and excitable properties including membrane resistance, capacitance and the amplitude of Na^+^ currents depending on brain area and reflecting their potent functional heterogeneity^2, 3, 5, 8, 25, 26^. In the MNTB, we found two classes of OPCs distinguished by the presence or absence of Na^+^ currents, as previously observed in the cerebellum and corpus callosum^2^. One class of OPCs exhibited Na^+^ currents and graded spikes with amplitudes that increased with increasing depolarization similar to OPCs in the visual cortex and corpus callosum^3, 8^. In the MNTB, OPCs had a larger input resistance and smaller membrane capacitance, reflecting a smaller cell size and exhibited a much smaller I_Na_ compared with excitable pre-OLs (Fig. 4). These differences in the physiological properties and the biophysical parameters of Na^+^ currents may underlie the less effective firing in OPCs.

Although excitable properties of pre-OLs in the MNTB differed from those of OPCs, both stages were comprised of two different subpopulations depending on the presence of I_Na_. This raises the possibility that excitable pre-OLs are derived from the class of excitable OPCs and conserve their excitability during differentiation and maturation. Previous studies suggested that OPC excitability rapidly decrease during the transition from OPCs to pre-OLs, and then finally disappear upon OL maturation^4, 8^. Here, the number of OPCs began to decrease at ~P7, whereas the population of excitable pre-OLs started to increase at ~P7, reached their peak at P13, and were maintained at a constant proportion into the beginning of adulthood (P31–P62). These results suggest that excitable pre-OLs do not have an ephemeral fate but rather functionally persist at least through the juvenile stage.

The presence of excitable pre-OLs throughout postnatal development raises the issue of their physiological role. One potential role is to regulate the structural maturation of OLs, because loss of their excitability in the Na_v_1.2 knockdown reduced glial processes and axon-glial synapses. Excitable pre-OLs are sensitive to neuronal activity and inputs. Thus, oligodendroglial excitability may mediate neuronal activity-dependent maturation of OL lineage cells and promote myelination during development^27, 28^. Unexpectedly, the majority of the CNPase-eGFP+ pre-OLs were affected by Na_v_1.2 knockdown, suggesting that Na_v_1.2 knockdown seems to have a broad effect on the population of shRNA-infected CNPase+ cells. We ruled out shRNA-related toxic effects and off-target effects using scrambled shRNA and two shRNAs targeting Na_v_1.2 at different sites. One possible explanation for the widespread effect of shRNA is that non-excitable OLs originally have Na_v_1.2 but rapidly lose the functional Na_v_1.2 channels during maturation. During OL differentiation, non-excitable pre-OLs may lose Na_v_1.2 channels and then rapidly proceed to myelination, whereas excitable pre-OLs with Na_v_1.2 channels may stay longer in the pre-myelination stage, not proceeding to myelination^4, 8^. Thus, the injection of a Na_v_1.2-specific shRNA at an early stage (P3) could impact Na_v_1.2 channel functions, i.e., increasing and extending processes, in both excitable and non-excitable pre-OLs. We did not find distinguishable Na_v_ currents and APs in non-excitable OLs; therefore, another possible explanation is that non-excitable pre-OLs may have too few Na_v_1.2 channels to generate distinct inward currents and APs. Thus, the presence of Na_v_1.2 in non-excitable pre-OLs may not contribute appreciably to their physiological properties or excitability, but the genetic modification of Na_v_1.2 could have sufficient effects on structural properties in non-excitable pre-OLs.

Yet another possibility is that excitable pre-OLs directly provide a critical signal to surrounding neurons and glia for maintaining axonal integrity and metabolism^29^. Na_v_1.2-mediated excitability may facilitate the paracrine effects of excitable-OLs on neighboring cells including non-excitable pre-OLs and axons. OPCs derived from embryonic stem cells secrete proteins (e.g., brain-derived neurotrophic factor) with the potential roles of enhancing neuronal survival and promoting axonal regeneration^30^. These neurotrophic factors could affect the maturation of both excitable and non-excitable pre-OLs^31^. A previous study using mutant mice lacking CNPase, displaying marked axonal swelling and progressive axonal loss, revealed that this axon pathology was due to loss of unknown signaling between OLs and axons^32^. This study suggests that OLs secrete signaling molecules to neighboring cells, including axons and glia. Additionally, CNPase+ OLs are electrically coupled among each other via gap junction channels, suggesting an important role of the inter-oligodendrocytic communication in myelin formation^33^. Excitable pre-OLs might provide a similar type of chemical and electrical signaling to facilitate maturation of surrounding pre-OLs. Therefore, loss of Na_v_1.2 in excitable pre-OLs could influence maturation of neighboring OLs including non-excitable pre-OLs.

In addition to the inter-oligodendrocytic communication, excitable pre-OLs could directly communicate with axons. Synaptic inputs from axons to OPCs have been found in the cerebellum, corpus callosum and cerebral cortex^4, 6, 8, 25^. OPCs express functional glutamate receptors and transporters and display AMPA/kainate receptor−mediated synaptic currents^34–38^. Here, we demonstrated physical and functional interactions between neurons and excitable pre-OLs, representing a more mature stage beyond OPCs. In the brainstem, excitable pre-OLs received glutamatergic inputs from surrounding axons or synapses and predominantly exhibited AMPA/kainate receptor−mediated currents, similar to OPCs in the cerebellum or hippocampus^39^. Brief stimulation of axon fibers can trigger synchronized synaptic currents in pre-OLs but may not recruit enough axons to provide sufficient glutamate for pre-OL depolarization. Under prolonged axonal stimulation or glutamate application, excitable pre-OLs showed Na_v_1.2-driven spikes, followed by slow and sustained depolarization mediated by AMPAR activation. There was a temporal delay between axonal stimulation and the onset of membrane depolarization. One possible explanation is that after strong stimulation, glutamate spillover from surrounding axons and synapses activates AMPARs and depolarizes pre-OLs. Another possibility is the presence of axonal activity-dependent volume transmission as well as rapid synaptic communication between axons and pre-OLs^40, 41^. Subsequently, the membrane potential reaches a threshold, and excitable pre-OLs are able to generate spikes. The slower non-synaptic communication mediated by glutamate has been observed previously, inducing a Ca^2+^ response that had an average time to peak of 25 ± 5.7 s after stimulation^41^. Another explanation is that the field stimulation can cause synchronous activation of axons and profound depolarization of surrounding glia, which increases extracellular K^+^^42^. As a result, there may be a wave of depolarization, upon which these spikes are indirectly induced. Under physiological conditions, the calyx of Held axons can routinely fire and propagate APs of >600 Hz; thus, the axon stimulation at 100 Hz is considered physiological.

It remains unclear why immature OLs from the same lineage develop differently in different brain regions and show distinct properties of excitability in the auditory nervous system. In the auditory brainstem, the number and length of processes were reduced by Na_v_1.2 knockdown in pre-OLs in both the MNTB and the midline region, where the heavily myelinated afferent fibers cross over. Thus, oligodendroglial Na_v_1.2 function is primarily associated with OL maturation and myelination in the auditory brainstem. In addition, we predict that in the MNTB, which is a synapse-rich area, the encompassing structures of pre-OLs surrounding the calyx synapses may contribute substantially to synaptic function. Satellite OLs, which are closely apposed to the neuronal soma, are involved in rapid uptake of extracellular K^+^ and assist neuronal high-frequency activity^42^. In the auditory brainstem, the excitability of OLs may be necessary for the temporal fidelity of auditory signals, which is a key element for the development of auditory processes and requires a high density of OLs and compact myelination^43^. During the last decade, different physiological properties and functions of OLs have been demonstrated depending on developmental stage and brain area. Considering the heterogeneity of OL lineage cells, our results contribute to our understanding of the physiology and function of immature OLs in the auditory brainstem.

## Methods

All procedures were carried out in accordance with National Institutes of Health guidelines and approved by the Institutional Animal Care and Use Committee of the University of Texas Health Science Center at San Antonio.

### Slice preparation

Transverse brainstem slices (200 μm thick) were prepared from Sprague-Dawley rats at P5−P62, representing different time points in OL development. After rapid decapitation, the brainstem was quickly removed from the skull and immersed in ice-cold low-calcium artificial cerebrospinal fluid (aCSF) containing: 125 mM NaCl, 2.5 mM KCl, 3 mM MgCl_2_, 0.1 mM CaCl_2_, 25 mM glucose, 25 mM NaHCO_3_, 1.25 mM NaH_2_PO_4_, 0.4 mM ascorbic acid, 3 mM myoinositol and 2 mM Na-pyruvate, pH 7.3–7.4 when bubbled with carbogen (95% O_2_/5% CO_2_), and 310–320 mOsmol/l. The brainstem was cut and the slices transferred to an incubation chamber containing normal aCSF bubbled with carbogen, in which they were maintained for 30 min at 35 °C and thereafter at room temperature (≤25 °C). Normal aCSF was the same as low-calcium (slicing) aCSF, but with 1 mM MgCl_2_ and 2 mM CaCl_2_. Sagittal slices of cerebellum (200 μm thick) were prepared from P9−P14 Sprague-Dawley rat pups using the same protocol.

### Electrophysiological recordings

Whole-cell patch-clamp recordings were performed in normal aCSF at room temperature (22–24 °C) using an EPC-10 amplifier (HEKA Elektronik) controlled by Patchmaster software. Voltage-clamp and current-clamp recordings of K^+^ currents and APs, respectively, were carried out using a pipette solution containing: 130 mM K-gluconate, 20 mM KCl, 5 mM Na_2_-phosphocreatine, 10 mM HEPES, 4 mM Mg-ATP, 0.2 mM EGTA and 0.3 mM GTP, pH 7.3 (adjusted with KOH). Voltage-clamp recordings to measure I_Na_ were carried out using a pipette solution containing: 130 mM Cs-methanesulphonate, 10 mM CsCl, 5 mM Na_2_-phosphocreatine, 10 mM HEPES, 4 mM Mg-ATP, 5 mM EGTA, 10 mM TEA-HCl and 0.3 mM GTP, pH 7.3 (adjusted with CsOH). Pipettes were pulled using an electrode puller (Model P-1000, Sutter Instruments) to open tip resistances of 5−6 MΩ. In all whole-cell recordings, Alexa 568 (40 µM; Invitrogen) was included in the pipette solution for post-recording labeling. Drugs used to induce or block synaptic currents included glutamate (1 mM; Sigma), CNQX (6-cyano-7-nitroquinoxaline-2,3-dione; 50 µm; TOCRIS) and Naspm (1-naphthyl acetyl spermine; 50 µm; TOCRIS).

### Immunostaining

Slices used for patch-clamp or fresh brainstem slices (~120 µm) were fixed with 4% (w/v) paraformaldehyde in phosphate-buffered saline (PBS) for 30 min. Free-floating sections were blocked in 4% goat serum and 0.3% (w/v) Triton X-100 in PBS for 1 h and then incubated with primary antibody overnight at 4 °C. The following primary antibodies were used: mouse anti-CNPase (1:200; Sigma, C5922), mouse anti-O1 (1:500; Millipore, MAB5540), mouse anti-Na_v_1.2 (1:50; Neuromab, 75-024 Clone K69/3), mouse anti-PLP/DM20 (1:100; Thermo Fisher, MA180034), mouse anti-NeuN (1:200; Millipore, MAB377), rabbit anti-GFAP (1:500; DAKO, Z033429), guinea pig anti-vGluT1 (1:1000; Millipore, AB5905), rabbit anti-NG2 chondroitin sulphate proteoglycan (1:50; Santa Cruz Biotechnology, sc-20162), mouse anti-MBP (1:500; BioLegend, SMI-99P) and rabbit anti-Na_v_1.6 (1:200, Alamone, ASC-009). Tissues were then incubated with different Alexa-conjugated secondary antibodies (1:500; Invitrogen) for 2 h at room temperature. After five rinses with PBS, slices were coverslipped using mounting medium with 4′,6-diamidino-2-phenylindole (DAPI; Vectashield; Vector Laboratories) to counterstain cell nuclei. Stained slices were viewed on a confocal laser-scanning microscope (Zeiss LSM-510 or Olympus IX81 Fluoview 1000) at 488, 568 and 633 nm using a 40× or 60× oil-immersion objective.

### Cell counting

Images of slices were acquired using a confocal microscope (IX81 Fluoview 1000) equipped with a 60× oil-immersion objective and appropriate filters for DAPI, Alexa 488 (Na_v_1.2), Alexa 568 (NG2) and Alexa 647 (CNPase). The oligodendroglial population was analyzed by capturing five random fields of the MNTB area (200 × 200 × 20 µm) per coverslip. Four replicates of each experiment (P5, 7, 9, 11, 13, 31 and 62) were performed, each with three coverslips per group. Oligodendroglial cells were counted as described^44^. OLs that exhibited clear DAPI labeling with NG2 and CNPase or Na_v_1.2 and CNPase were counted as positive or double-positive cells.

### Vector construction and virus production

A cDNA encoding the CNPase promoter sequence and the eGFP reporter gene (pAV.ExSi-CNPase promoter-eGFP) was produced and inserted into an adenovirus packaging vector. Briefly, the adenoviral vector was linearized and transfected into optimized packaging cells. Packaged virus was collected from infected cells and used to infect additional packaging cells to further amplify the virus. To assess the role of Na_v_1.2, we used an adenovirus with the same CNPase promoter sequence with an additional sequence encoding shRNA targeting the gene *SCN2A* (encoding the Na_v_1.2 channel) as well as an eGFP reporter (Supplementary Table 1). Adenovirus containing vector only (control), the scrambled shRNA, and shRNA1 and shRNA2 were injected into the MNTB in the auditory brainstem using stereotaxic injection. Adenoviruses carrying each of these plasmids were purchased from Cyagen Biosciences.

### Stereotaxic injection

Sprague-Dawley rats were injected at P3 with the adenovirus carrying the appropriate plasmid in the MNTB. Rats were anaesthetized on ice (10 min; to avoid harmful effects of isoflurane) and maintained one ice throughout the surgical procedure. The animals were placed in a stereotaxic frame (David Kopf Instruments), the scalp was opened, and the lambda relative to bregma was measured. Typical coordinates for injection were (in mm, from bregma) A/P –4.8, D/V –6.5, M/L 0.5. Adenovirus containing the appropriate plasmid (1 μl at >10^12^ particles per ml) was injected unilaterally using a 30 G injector (Plastics One) at a rate of 0.25 µl/min. The needle was allowed to remain in place for 2 min and then slowly removed. The scalp was glued using tissue adhesive (3 M Vetbond), and all traces of blood were removed. Animals were removed from the stereotaxic frame and placed in clean cages under light at 37 °C. After full recovery, rats were returned to their respective cages. Animals were killed (as describe in the slicing section) at 7–14 days after the injection to obtain brain slices.

### Western blotting

Virus-infected brainstem slices (200 μm), displaying CNPase-GFP+cells, were homogenized using a protein extraction buffer (ThermoFisher Scientific) as well as protease inhibitor cocktail. The lysates were incubated for 30 min on ice and then centrifuged at 15,000 r.p.m. for 30 min at 4 °C. Supernatants were collected and protein concentrations were estimated using a BCA protein assay kit (Thermo Scientific). Equal amounts of protein were resolved by 12% sodium dodecyl sulfate–polyacrylamide gel electrophoresis gel and transferred onto polyvinylidine fluoride membrane. The membranes were blocked for 1 h at room temperature and incubated overnight in primary antibody (MBP, 1:1000, BioLegend, SMI-99P and β-actin, 1:1000, Cell signaling, 8H10D10) at 4 °C. Membranes were incubated with IR-conjugated secondary antibodies for 2 h and scanned using Li-COR Odyssey IR imager. MBP band intensities were quantified and normalized to β-actin. Images of Western blots have been cropped for presentation. Full-size western blots with protein ladders (LI-COR, P/N 928-60000) are shown in Supplementary Fig. 7.

### Data availability

The authors declare that all data generated or analyzed in this study are available within the article and its Supplementary Information files. The data that support the findings of this study are available from the corresponding author upon reasonable request.

## Electronic supplementary material

Supplementary Information
