# Supplementary material for: Oligodendroglial excitability mediated by glutamatergic inputs and Nav1.2 activation
Source: Nat Commun. 2017 Sep 15;8:557. doi: 10.1038/s41467-017-00688-0 (PMC5601459; doi:10.1038/s41467-017-00688-0)
Supplement: Supplementary file 1 — Supplementary Information [file 41467_2017_688_MOESM1_ESM.pdf]

Description of Supplementary Files

File Name: Supplementary Information
Description: Supplementary Figures and Supplementary Table.

Supplementary Figure 1

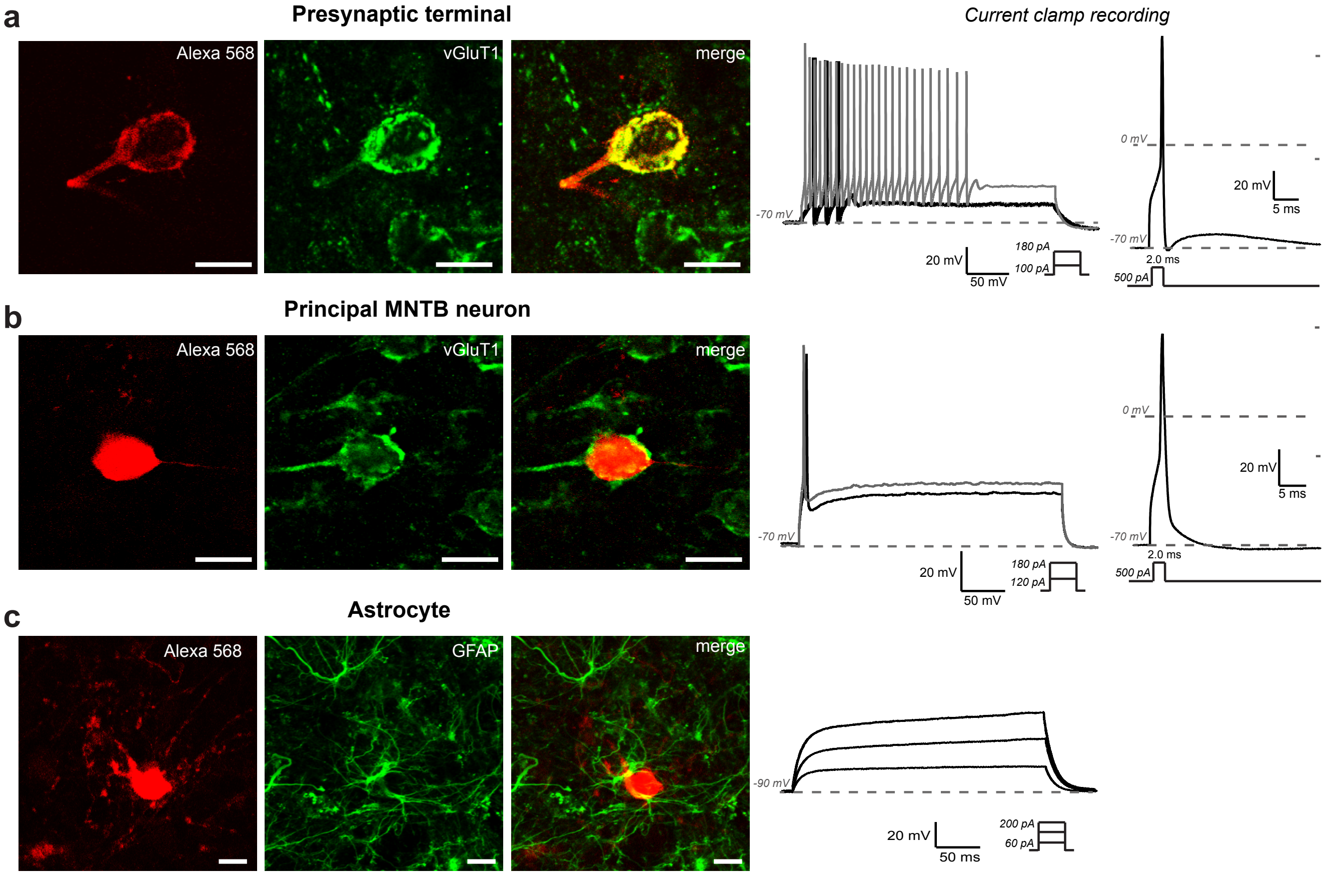

Supplementary Figure 1: Excitable properties of different cell types in the MNTB. (a) Post-recording immunostaining of the MNTB calyx of Held presynaptic terminal loaded with Alexa 568 (red) and stained with vGluT1 (presynaptic marker; green) at P12. Scale bar: 20 μm. Step-current injection (100 to 180 pA; 300 ms) induced repetitive AP firing activity, where the number of APs increased with the size of the injected current. A single AP was generated by a brief current injection (500 pA, 2.0 ms; left). APs displayed a marked threshold and profound after hyperpolarization typical of calyx of Held terminals. **(b)** Post-recording immunostaining of MNTB principal neuron loaded with Alexa 568 (red) and stained with vGluT1 (green) at P13. Scale bar: 20 μm. Step-current injection (100 to 180 pA; 300 ms) induced single AP firing activity typical of principal MNTB neurons. A single AP was generated by a brief current injection (500 pA, 2.0 ms; left). APs displayed a marked threshold, but no AHP, and were wider than presynaptic APs. **(c)** Post-recording immunostaining of MNTB astrocyte loaded with Alexa 568 (red) and stained with GFAP (astrocyte marker; green) at P12. Scale bar: 10 μm. Step-current injection (60 to 200 pA; 300 ms) did not induce activity. All recordable cells in the MNTB (calyx of Held terminal, MNTB principal neuron, and astrocytes) displayed different shapes, staining patterns, and firing properties than excitable OLs.

Supplementary Figure 2

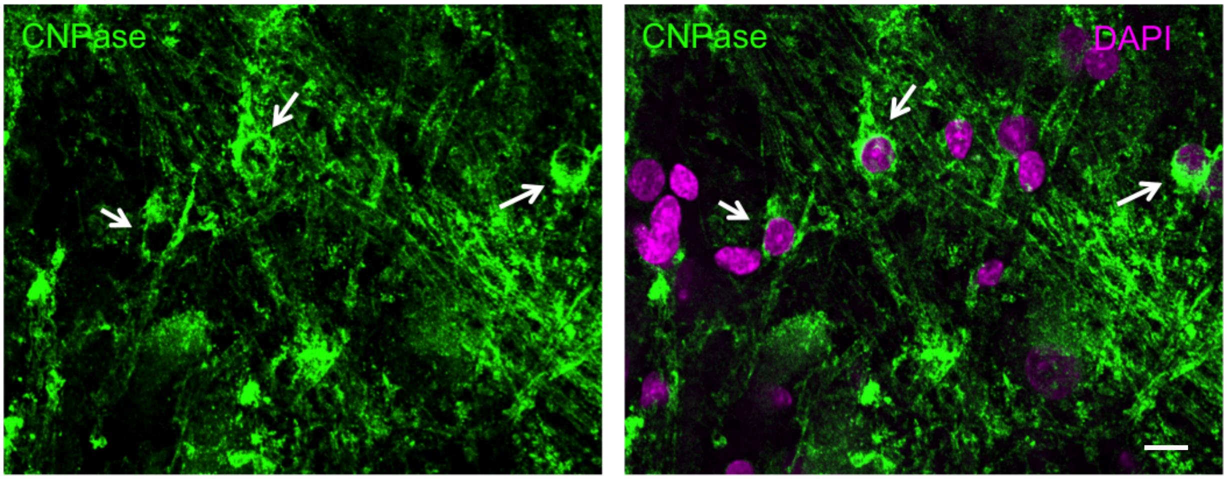

Supplementary Figure 2: CNPase immunoactivity was strongly detected from processes and somatic membrane of pre-OLs. Immunostaining for CNPase (green) and DAPI (blue) in the MNTB (P13). Scale bar: 10 μm.

Supplementary Figure 3

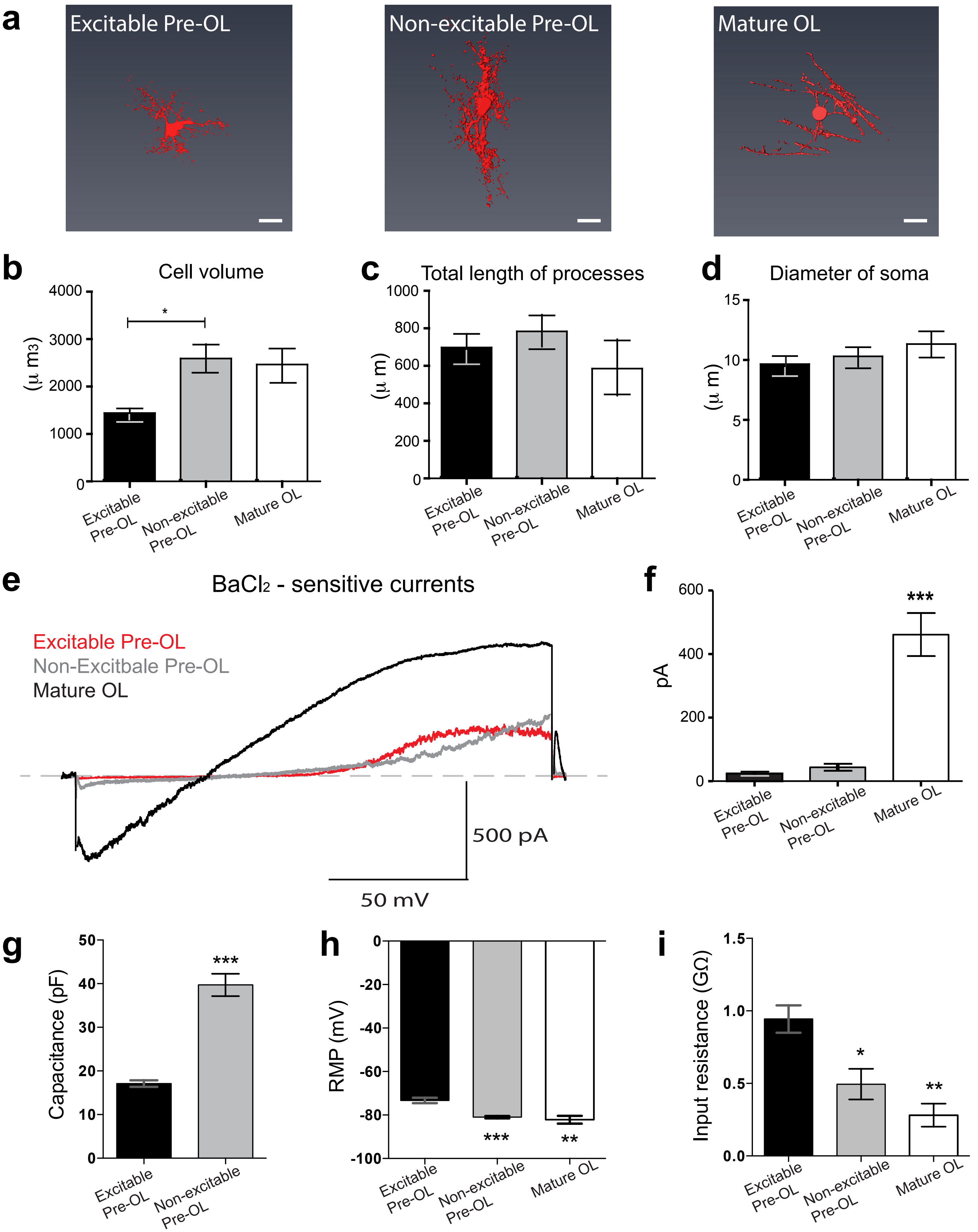

Supplementary Figure 3: Extended comparison of the physiological and morphological properties of excitable pre-OLs, non-excitable pre-OLs, and mature OLs. (a) 3D reconstruction of dye-filled excitable pre-OL, non-excitable pre-OL, and mature OL. Scale bar: 20 μm. **(b-d)** Morphological properties of excitable pre-OLs, non-excitable pre-OLs, and mature OLs: cell volume **(b)**, total process length **(c)**, and soma diameter **(d)**. Data presented as mean ± s.e.m.; $*p < 0.05$. **(e)** Representative traces of Ba+-sensitive currents in response to a ramp depolarization in each of the OL types. Traces acquired by subtracting the current after the addition of 1 mM $BaCl_2$ from the current before the addition. **(f)** Ba+-sensitive inward current amplitude in each OL type. **(g-i)** Comparison of passive properties – membrane capacitance **(g)**, input resistance **(h)**, and resting membrane potential (RMP) **(i)** – in three different subpopulations of OLs. Data presented as mean ± s.e.m.; $*p < 0.05$; $***p < 0.001$.

Supplementary Figure 4

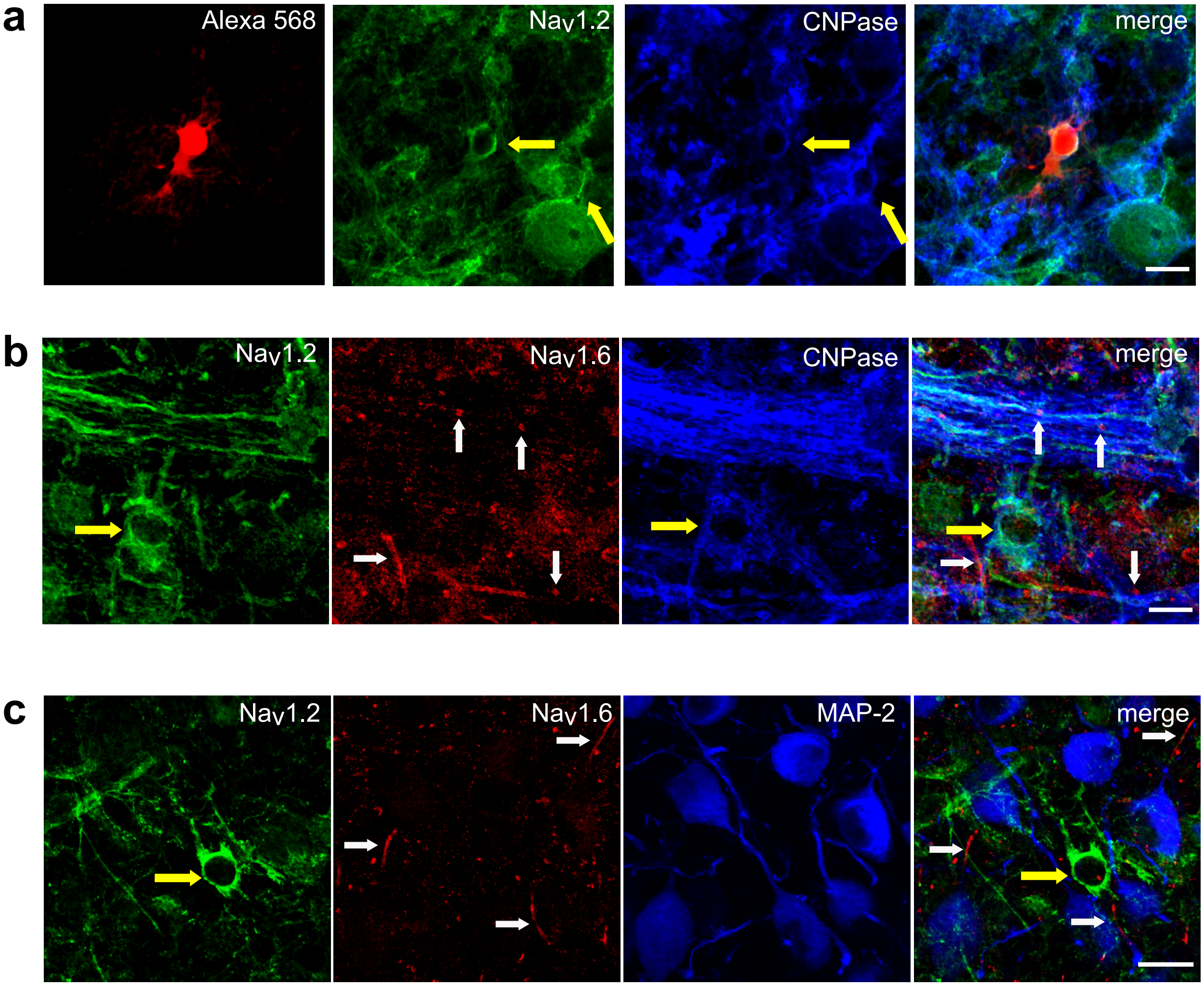

Supplementary Figure 4: Nav1.2 expression pattern is distinct from Nav1.6 expression. **(a)** Expression of Nav1.2 and CNPase in excitable pre-OLs (red arrow). CNPase+ pre-OLs located close to the MNTB neuron (asterisk) and their processes covered the neuronal soma (yellow arrow). Scale bar: 20 µm. **(b)** Immunostaining for Nav1.2 (green), Nav1.6 (red), and CNPase (pre-OLs, blue) in the MNTB (P13). Nav1.2 was expressed in pre-OLs (co-staining with CNPase, yellow arrow). Nav1.6 was mostly located at nodes of Ranvier and the axon initial segment (AIS, white arrows). There was no detectible Nav1.6 signal in pre-OLs (CNPase+ cells). Scale bar: 10 µm. **(c)** Immunostaining for Nav1.2 (green), Nav1.6 (red), and MAP2 (principal neurons, blue) in the MNTB (P13). Nav1.6 was located at the AIS of MNTB neurons (white arrows). Scale bar: 20 µm.

Supplementary Figure 5

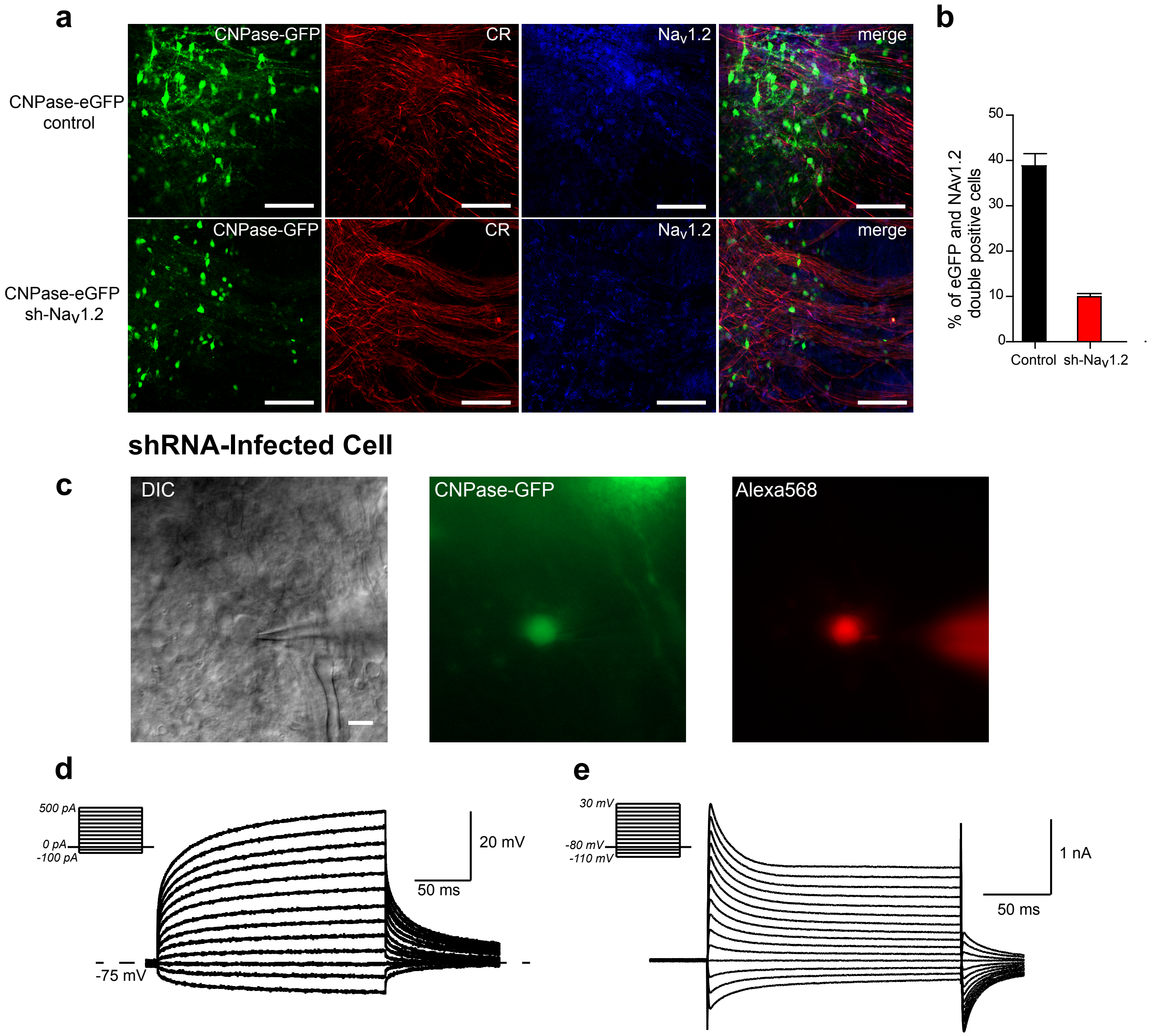

Supplementary Figure 5: Nav1.2 knockdown affects pre-OL morphology and physiology. (a) Immunostaining of CNPase-eGFP+ cells, calretinin (red) and Nav1.2 (blue) in the MNTB (at P21) in control condition (upper panel) and following the injection of shRNA for Nav1.2 (lower panel) at 20X magnification. Scale bar: 100 µm. **(b)** Bar graph reporting the proportion (in %) of the double positive cells for CNPase-eGFP and Nav1.2 signals. **(c)** CNPase-GFP+ cells from animals injected with the shRNA virus were recorded using whole-cell patch clamp electrophysiology and filled with Alexa 568. Scale bar: 10 µm. **(d)** shRNA-infected cells displayed no action potential in response to depolarizing current injections. **(e)** shRNA-infected cells displayed no INa in response to voltage injections.

Supplementary Figure 6

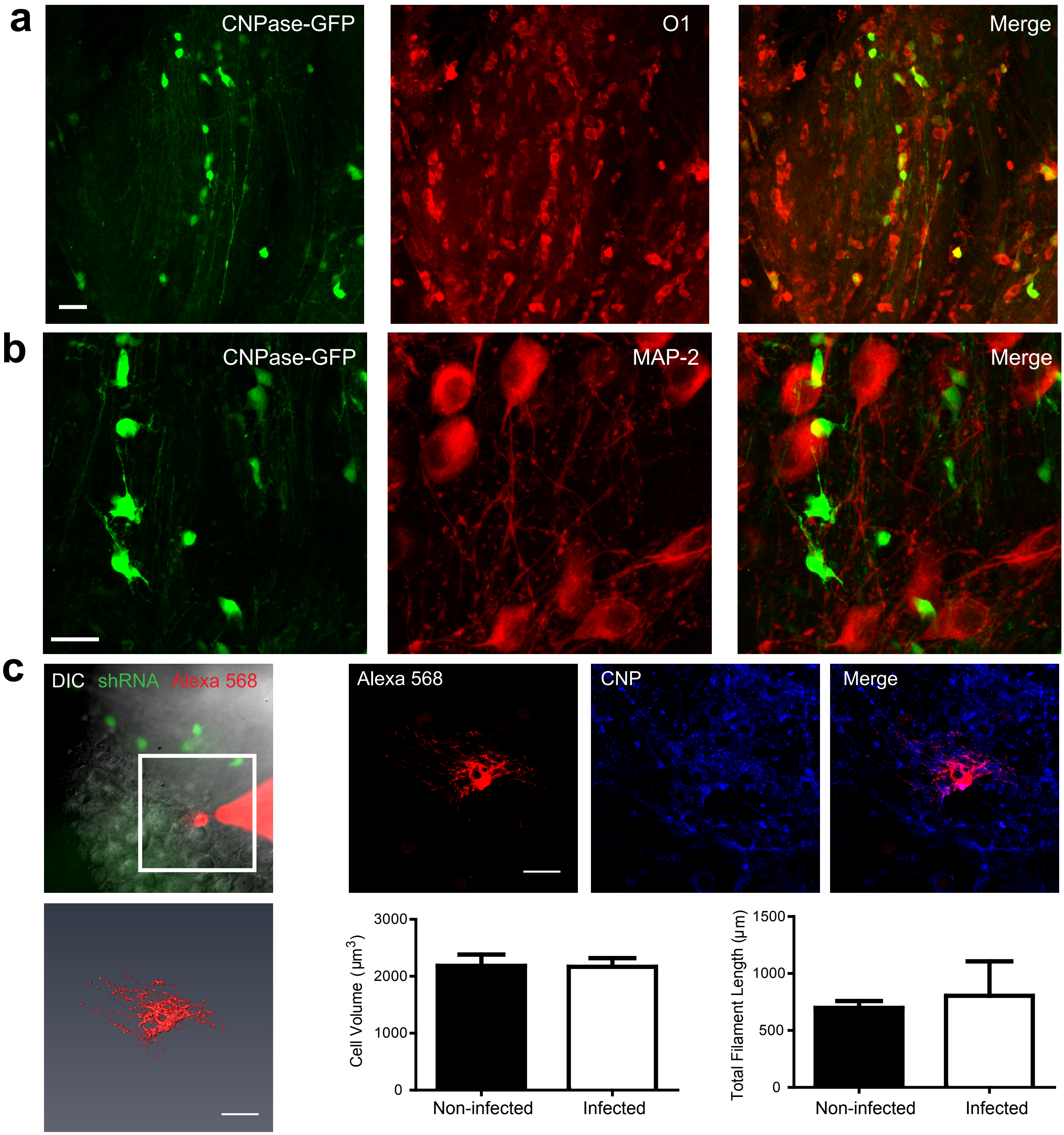

Supplementary Figure 6: Specific and direct targeting of shRNA on CNPase-GFP-expressing cells. **(a)** Immunostaining of CNPase-eGFP+ cells (green) and O1 (OL marker; red) in the MNTB. Scale bar: 50 μm. **(b)** Immunostaining of CNPase-eGFP+ cells (green) and MAP2 (neuronal marker; red) in the MNTB. Scale bar: 10 μm. **(c)** GFP-negative pre-OLs in the MNTB were subjected to whole-cell recording and were filled with Alexa 568 (red) during recording for post-recording immunostaining. (upper left panel) Differential interference contrast (DIC) image of a GFP-negative pre-OL, which was loaded with Alexa 568 (red). (upper right panel) Post-recording immunostaining of Alexa 568–filled GFP-negative pre-OL (red) and CNPase (blue) in the MNTB. Scale bar: 20 μm. (lower left panel) 3D reconstruction of dye-filled GFP-negative pre-OL. Scale bar: 20 μm. (lower middle and right panel) Morphological properties of GFP-negative pre-OL: cell volume (lower middle panel), total process length (lower right panel). Note: dye-filled OLs that did not express GFP, had no alterations in their morphology compared to wild-type dye filled cells. The shRNA have no indirect effect, and work solely on the cells that express GFP.

Supplementary Figure 7

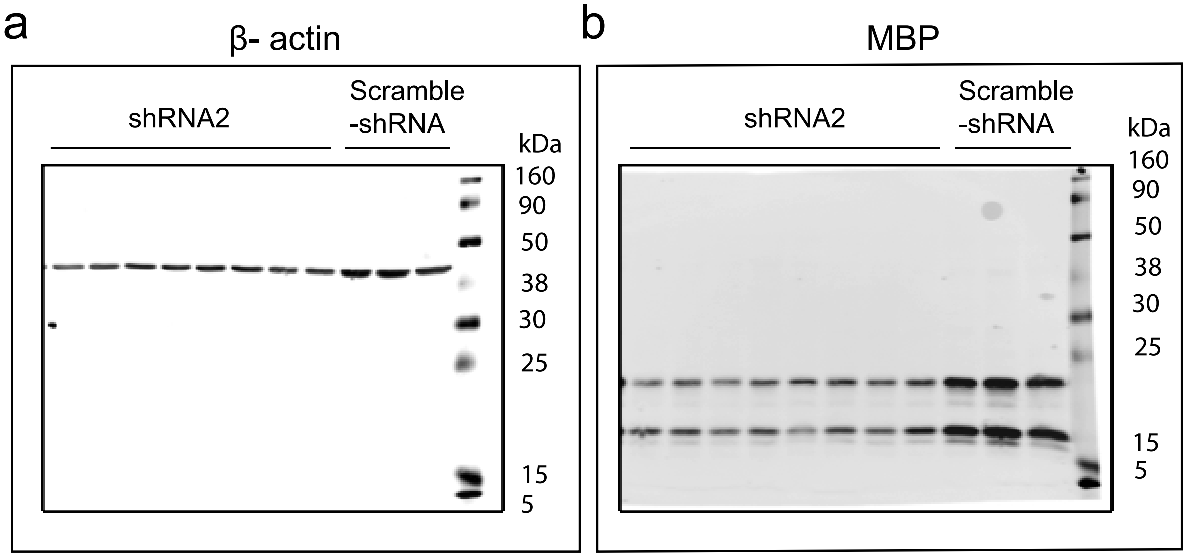

Supplementary Figure 7: Full membrane for Western blot for β-actin (a) and MBP (b) in the shRNA virus- and scrambled shRNA virus-infected groups. Blot shows the corresponding expected band at 45kDa for β-actin and at 18, 21.5kDa for MBP.

Supplementary Table 1

CNPase promoter sequence:	TCTAGAACAGCACAGAAATCCACTGAGAAGGCTGTGACTTCCTAGAGTTAGCGAATAAACATCTGGCCT<br>CAAAAGGATCAGCCTGGGCTGGAGAGATGGCTCAGTGGTTAACAGCAGTCCTGAGTTCAATTCCCAGC<br>AACCATGTGGTGGCTCACAACTGTCTGTAATGGGATCTGATGCCCTCTTCTAGTGGATCTGAAAACAGC<br>AACAGTGTTCTCATACACATAAAATAAATAAATAAATCTGGAAAAAAAAAAGATCAGGCCATCTTAGGGTT<br>TTAACTACTGTGAACAGACACCGTGACCAAGGCAACTCTTAGACAACATTTAATTGGGGATGGTGGTTTA<br>CAGGTTCAGACGATTATTATTAAGGTGGGATTGTGGCAGAATCCAGGCAGGCTGGAGGACCTGAGAGT<br>TCTATATCTTCATCTGAAGGCTGCTATGAGAAGACTGACTTCCAGGAAGCTAGAATGAGGGTCTTAAAGC<br>CCATGTCCACAGTGACACACTTTCTCCAACAGAGTCACACCTCCTAATGGTGCCACACCCTGGGCCAA<br>GCATATACAAACCATCATGCAAGGCCTTTGCCATGTGGTCCAGAAGCAGGGATAAAGATGACTTAGGCT<br>GGATGAAGCCAGGATTCCAGTCAAGTGAGAGGCAATTCAAAGGCAGAATGACTTGGTCCCCAAACTTAT<br>CCCCAGGTCAAGTCCAGGAACTTGTGCCTACTAGACAAGTCTTCTAACACCAAGCTATAGTCCCAGTCC<br>CTAGATAAAAGTATTTTAAATCATGTCTTTTTAAAATTACATTTTAAATGAGGCCGGAGAGATAGCGCAGAG<br>GCTAAGAGCAAGGGCTGCTCTTTAAGAGGACCTGAGTTCAATTCCCAGCAACCACATGGCAGCTCACA<br>CCTGTCTATAACTCTAGTTCCAGAAGGTTCTAAACCCCCACATAGACACATATGCAGGCGAAACACCAAT<br>ACACATAAAATAAAACTCATCTATTAAGCCAGGCAGTGGTGGTACACATTGTTAGTCCCAGCATCAGGGA<br>GGCAGAGGCAGCCGGATCTCTGTGAGTTTGAGGCCAGCCTGGTCTACACAGTGAATTCAGGATAGCCA<br>GAGCTGGACAGAGAGATCTCTCAAAGAAATAAAAAGTACATTTTAATTTATTTTTGTGTGCATTTATGTGT<br>GCGGACCTACACACCTACTACCTGCCTGTGGTGTCAGAGAAGAACAAGTCGAGGGAGTTGGTTTTCTA<br>TCATTCATGGCCTAGAAGTTCAACTCAGGGTAACGTGGCCATGTTAACAAAGCTATATCTCAACTGCTCC<br>TGAGAAATTATATATAATTATATATCCGTTTGTTTTGTTTTGTTTTGTTGAGACAGGGTGTCTCTCTGTAGGC<br>CTGGCTGTCCTGGAACTCACTCTGCAGATCAGGCTGGCCTGGAACTCACTGAGATCTGCTTGCCTCTG<br>CCTCCCAAGTGCTGGGATTAAAGAAAAAAGTGAAAGTGAACAATGTCACCACGTCCTCGAAGCTAGGAT<br>GCACTTGCCTAGCTTGCAGGAACCCCAGCATCTTAAACAGCTGACATGTTCTTGTATGTATGAATTTCCA<br>GCAAGAGGATAAGAAGTTCAAGGTCACCCTTTGTTACATAGAGAGTTTGAACTTAGCCTGGGTTACATGA<br>GATTCATCTTTAAAACAAACCTTTTTTGGGGATATCCAGCAAAGAAGACTGCTTAGACCCAAGCGGAAGC<br>AATAGAAAGTCTTTATTAGCCAGCCCCTCAGCCCCTTCACTAGACTTTTTTTTTTTTTTTTTTTTTTTCCTG<br>AGACAGGGTTTCTCTGTGTAGACCTGGCTGTCCTGGAACTCACCCTGTAGACCAGGCTGGCCTTGAAC<br>TCAGAAATCCACCTGCCTCTGCCTTCCAAGTGCTGGGATTAAAGGTGTGTGCCACCACCACCCAGCAC<br>TTTTCTCAGGGTAGGCTTTTAATCATAAAAACCACATCCTGTGTTGACACACCTAAGTCAGAAGCAGACT<br>ATAGGAGCCAAAAATCAAGGTTAGGACATTTAGGGCCTTTCCCAGAATTATGGACTTTGATGAATTAGGT<br>CTTGGTTTTGGTTTAGGAGGCGGTGATGCCTATCGGCCTGGTTTCGTGSCCTGAATGGTACTTCCTTCA<br>CATGTCAGTTGTGATAAGGTCTATGAGCCTGTTTCAAAATTTTAAACTATCTGACTGCACCCATTTCATCC<br>CAGCCTGCCTGGGAACCCAGGAAAGGCAACTGCCTCCTGTTTTAAGAGTTCATCTCTCTCCTGGTGAT<br>GTCTTTAATCCAAGCACTTGGGAGGCAGAGGCAGGCAGATCTCTGTACCAGGCCAGCCTGATCTACATG<br>GTGAGTTCCAGGACAGTCAGGGCTACATAGTGAGTTCCTGTCTCAGAGAAGGGAAAAAGTTAAACTTTA<br>ACTTTTAAGGAACAAAGAGGTGGCGTGGCGGACTCTTTCTTGGCATTGAGAGTCCAGGTGTGCTTTTAC<br>TTTCTGCCAAGGCTCAGACCGCTGCTTCCTGGCACTCAGGTTTCCATTCCCCGGGGGCGCGTGCGCA<br>GAGGCTCGGGCTGACCCCGCCCTGTCCGCGCGCCCCGCCCCCAGGCTATGAAAGGGACCCTGCGTG<br>GGGGGGCCCCGGACACCCGGAGACGCCCGTGTCCCTCACGCAGGCGGGCGGCCCCGGAGACATAG<br>TACCCGCAAAGGCGGTGACGGCGGTGCGCCCACTCATCATGGTGAGCGCAGCGCGTGCGTGGGGGC<br>CGGGGCGGGAGAGAGGCTAACTGAAAGCGAGATTCTTGGGGATGCATGGGCAGAAGTAGTTTGCAAAT<br>GAGGACACCAGGTTCCGGAGTTGAAGTTGCCTCTGGGTCCTCCAAGGCAGCCCTAAGGCAAGGGGCG<br>CGAGCGGGGATATTTTGGGGGAGTCTTAGTGTCTTTGGAGAAGGAAATATCGCCGCAGGAAGTGAAAG<br>TCGATGCTCCTTGGCGACACCAGCTGGCAAATGGGTCTGGGTAGCACCAGAGAGTCGAGGGCTGTTG<br>TGGAGCCCGCGCTGGGAGTGGGTTCCTGCACAAGGCTATAGAGAAGCCAGAAGCCAGCTAAATTCTCA<br>GGGGCGGAAGCCGTTGTAGATGACAACTGAAACCGCTATGTCTTGTTTTTGTCAGAGAGGTGCTGCTAA<br>CACACAGCAGAATAGGGGGCACAGATCTCTGAATACAGGTGCCCTGTGCCCTAATCCTACAGATTGGGG<br>GAGGGGGCGCACTGAGAGAGCAGAGGAGGAGGTGGTTGTGAATATGCCCTTTGTCCAGTGCCCTCCT<br>GCCCTGCCCCCCACCAGCCCTCTGTGGGACCATTGTCCCCCTATACCCAGACAAGAGAGTATTATCTGT<br>TGGTGCCATTGTTGGGTGGAGGGAGGCGGTCTTTGGGTCACCCTCCCCTGCCCTCACCCTCCTCTGC<br>ACACTCAAGAGAGCCAAGGCAGCTGTGGCTTTGCCCATACATAGAGCCCCAGGCCTCCAAACAGGACA<br>TCCTTAAGAGGTGCCAAGAATATATAAGTTAGGGCTGACAGGGAGCCACAGGCACTTGCTGCCCTTGCG<br>TGCCCTGTCACCAAGGTGTTCTGAGGGGGAGCAGAGAGTGATCCTTGGAGCCAGAGACTAAGAGGTTA<br>GGTAGCTTCAGAAAGATTCCCTCTACCTTACAACCCAGCTGTTGAAGATACTGAAGGATCTACTGCGAGG<br>AAGAAAATCCACCATTCCCCTCCGGGGAAAGCATCCACCATTCAAGGGTGCTTTAAGTTAGGCTTGGGG<br>ATTCCTCAACTGACAAGCGGTCCCCCTCTGGGAGAACTCGGCCAGAGACTAGGGTGTGGTGCTTGCG<br>GGCTGGCTTTGAGGAGCCGGGGCTGACATCTCCTCCTCCCTTCTTACACAGAACACAAGCTT
shRNA1 sequence:	TGTCAAATATGAGCTCACAATGC
shRNA2 sequence:	TCATAAACACACAGTTGGTAAG
Scrambled shRNA sequence:	CCTAAGGTTAAGTCGCCCTCG

Supplementary Table 1: Nucleotidic sequences of CNPase promoter and the shRNA used in the vector.
